# Paediatric proteomic signatures of cardiometabolic disease-associated traits predict adult disease outcomes

**DOI:** 10.1038/s42255-026-01589-7

**Published:** 2026-09-11

**Authors:** Joshua M. Landman, Heather M. Highland, Andrew S. Perry, Annie G. Howard, Quanhu Sheng, Anna Lorenz, Alexandra B. Palmer, Shilin Zhao, Wanying Zhu, Xinruo Zhang, Victoria L. Buchanan, Elizabeth G. Frankel, Rashedeh Roshani, Alyssa Scartozzi, Eric H. Farber-Eger, Mohammad Y. Anwar, Jessica K. Sprinkles, Ash Breidenbach, Ting-Chen Wang, Christine A. Ballard, Matthew Nayor, Jaclyn Tamaroff, Absalon Gutierrez, Lauren E. Petty, Alexander S. Petty, Benjamin Lippi, Lindsay Fernandez-Rhodes, Hung-Hsin Chen, Mohanraj Krishnan, Mariaelisa Graff, Katie A. Meyer, Miryoung Lee, Kristin L. Young, Quinn Wells, Kahraman Tanriverdi, Jane E. Freedman, Eric R. Gamazon, Joseph B. McCormick, Susan P. Fisher-Hoch, Penny Gordon-Larsen, Jennifer E. Below, Kari E. North, Ravi V. Shah

**Affiliations:** 1 Vanderbilt Genetics Institute, Vanderbilt University Medical Center, Nashville, TN USA; 2 Division of Genetic Medicine and Clinical Pharmacology, Department of Medicine, Vanderbilt University Medical Center, Nashville, TN USA; 3 Department of Biomedical Informatics, Vanderbilt University Medical Center, Nashville, TN USA; 4 Department of Epidemiology, Border Health Research Center, School of Public Health, Brownsville Regional Campus, The University of Texas Health Science Center at Houston, Brownsville, TX USA; 5 Department of Epidemiology, Gillings School of Global Public Health, The University of North Carolina at Chapel Hill, Chapel Hill, NC USA; 6 Vanderbilt Translational and Clinical Cardiovascular Research Center, Vanderbilt University Medical Center, Nashville, TN USA; 7 Department of Biostatistics, Gillings School of Global Public Health, The University of North Carolina at Chapel Hill, Chapel Hill, NC USA; 8 Department of Biostatistics, Vanderbilt University Medical Center, Nashville, TN USA; 9 Division of Genetic Medicine and Clinical Pharmacology, Department of Medicine, Vanderbilt University School of Medicine, Nashville, TN USA; 10 Vanderbilt Institute for Clinical and Translational Research, Vanderbilt University, Nashville, TN USA; 11 Department of Nutrition, Gillings School of Global Public Health, The University of North Carolina at Chapel Hill, Chapel Hill, NC USA; 12 Sections of Cardiovascular Medicine, Department of Medicine, Boston University Chobanian & Avedisian School of Medicine, Boston, MA USA; 13 Division of Pediatric Endocrinology and Diabetes, Department of Pediatrics, Vanderbilt University Medical Center, Nashville, TN USA; 14 Division of Endocrinology, Diabetes and Metabolism, Department of Internal Medicine, The University of Texas Health Science Center at Houston, Houston, TX USA; 15 Department of Biobehavioral Health, College of Human and Health Development, Pennsylvania State University, University Park, PA USA; 16 Institute of Biomedical Sciences, Academia Sinica, Taipei, Taiwan; 17 Nutrition Research Institute, The University of North Carolina at Chapel Hill, Chapel Hill, NC USA; 18 Division of Cardiovascular Medicine, Department of Medicine, Vanderbilt University Medical Center, Nashville, TN USA; 19 Department of Medicine, Vanderbilt University Medical Center, Nashville, TN USA; 20 Vanderbilt Diabetes Center, Vanderbilt University Medical Center, Nashville, TN USA

**Keywords:** Predictive markers, Obesity, Metabolism, Proteomic analysis, Metabolic disorders

## Abstract

While the earliest pathological signs of cardiovascular–kidney–metabolic disease (CKMD) emerge before age 20, current adult-based risk thresholds fail to identify a substantial fraction of children at high risk. With increasing childhood obesity, early, sensitive detection of CKMD risk is critical to timely intervention. We linked 25 CKMD phenotypes spanning liver, adipose, vascular and dysglycaemia traits to the circulating proteome in 273 Hispanic or Latino children and adolescents (13.1 ± 2.7 years; 53% female). Here we show that proteome signatures of CKMD in children are strongly associated with clinical CKMD outcomes in adults. In 685 adults from the same community and 28,256 adults from UK Biobank, we observe high concordance between the paediatric and adult CKMD phenotype–proteome relationships, related to pancreatic beta-cell health and insulin sensitivity, liver homeostasis, inflammation and cholesterol metabolism. Many proteins linked to paediatric CKMD were modifiable with glucagon-like peptide-1 receptor agonist therapy in adults and are linked to genetic liability to CKMD. These findings support an early origin of CKMD disease liability embedded in the human proteome in children, underscoring the value of precision prevention to forestall metabolic disease long before its clinical manifestation.

## Abstract (web summary)

By linking cardiovascular–kidney–metabolic disease (CKMD) phenotypes to the proteome of children and adolescents, the authors show that paediatric CKMD proteomic signatures predict cardiometabolic disease outcomes in adulthood.

## Main

The earliest pathological signs of cardiovascular–kidney–metabolic disease (CKMD) emerge before age 20 (ref. ^1^), driven by cumulative exposure to obesity, insulin resistance, hypertension and pro-atherogenic dyslipidaemia^2^. Despite strong epidemiologic evidence highlighting the importance of these risks to premature CKMD^3–8^, current CKMD prevention efforts remain squarely focused on adults, missing an opportunity to identify high-risk individuals at the earliest stage of childhood. While most children and adolescents harbour intermediate subclinical phenotypes that fall below adult thresholds, even ‘normal’ values in adolescents are linked to CKMD in adulthood^3,4^. In addition, screening tests commonly used in adults (for example, fasting glucose) miss a substantial fraction (up to 70%)^9^ of dysglycaemia and type 2 diabetes (T2D) in children^10^. As a response, molecular biomarkers have been advanced in adults as a ‘precision medicine’ approach to detect disease liability decades before clinical onset^11–14^, although analogous studies in paediatric populations are less mature. A key challenge is that identifying paediatric biomarkers to predict clinical risk requires decades-long follow-up data, limiting efforts to identify reversible, actionable, prognostic mechanisms in childhood.

In this Article, we linked 25 CKMD phenotypes in 273 Hispanic or Latino children and adolescents (mean age 13.1 ± 2.7 years; 53% female) to the circulating proteome to identify molecular biomarkers of early CKMD susceptibility in children. CKMD phenotypes spanned clinical and imaging-based measures known to influence risk in adults, including body composition, liver fat and fibrosis, renal function and traditional CKMD risk factors. We identified proteomic patterns of these phenotypes in children at single- and multi-trait levels, generating multi-CKMD phenotype signatures to capture shared molecular risk, high interrelatedness, frequent co-occurrence and shared mechanistic pathways. We subsequently mapped the paediatric multi-protein composite CKMD signatures to adult CKMD outcomes in two samples: (1) 494 Hispanic or Latino adults from the same community (mean age 51 ± 14.3 years; 68% female) and (2) 28,256 adults from the UK Biobank (median age 58 years; 54% female). Given the application of glucagon-like peptide-1 receptor agonist (GLP-1RA) therapy in adults and children, we evaluated whether proteins implicated in paediatric CKMD were modifiable during GLP-1RA therapy in adults in the Semaglutide Treatment Effect in People with Obesity (STEP) 1 randomized clinical trial^15^. In parallel, we constructed genetic instruments^16^ of the circulating proteome to conduct proteome-wide association studies (PWAS) to test whether this GLP-1RA-responsive paediatric CKMD proteome would show a potentially causal association with adult CKMD outcomes (such as obesity, T2D, hepatic steatosis, myocardial infarction and kidney function). Finally, we delineated sources of variability in the paediatric CKMD proteome, as observed in recently published resources^17^, as understanding such heterogeneity is a prerequisite to targeted intervention. Ultimately, our goal was to map the proteomics of multidimensional CKMD-risk phenotypes in childhood with adult clinical CKMD markers through deep phenotyping and proteomic and genomic analyses, thereby offering a potential precision prevention approach to address clinical CKMD at its earliest manifestation.

## Results

### Characteristics of paediatric and adult samples for single-trait analyses

**Fig. 1:**
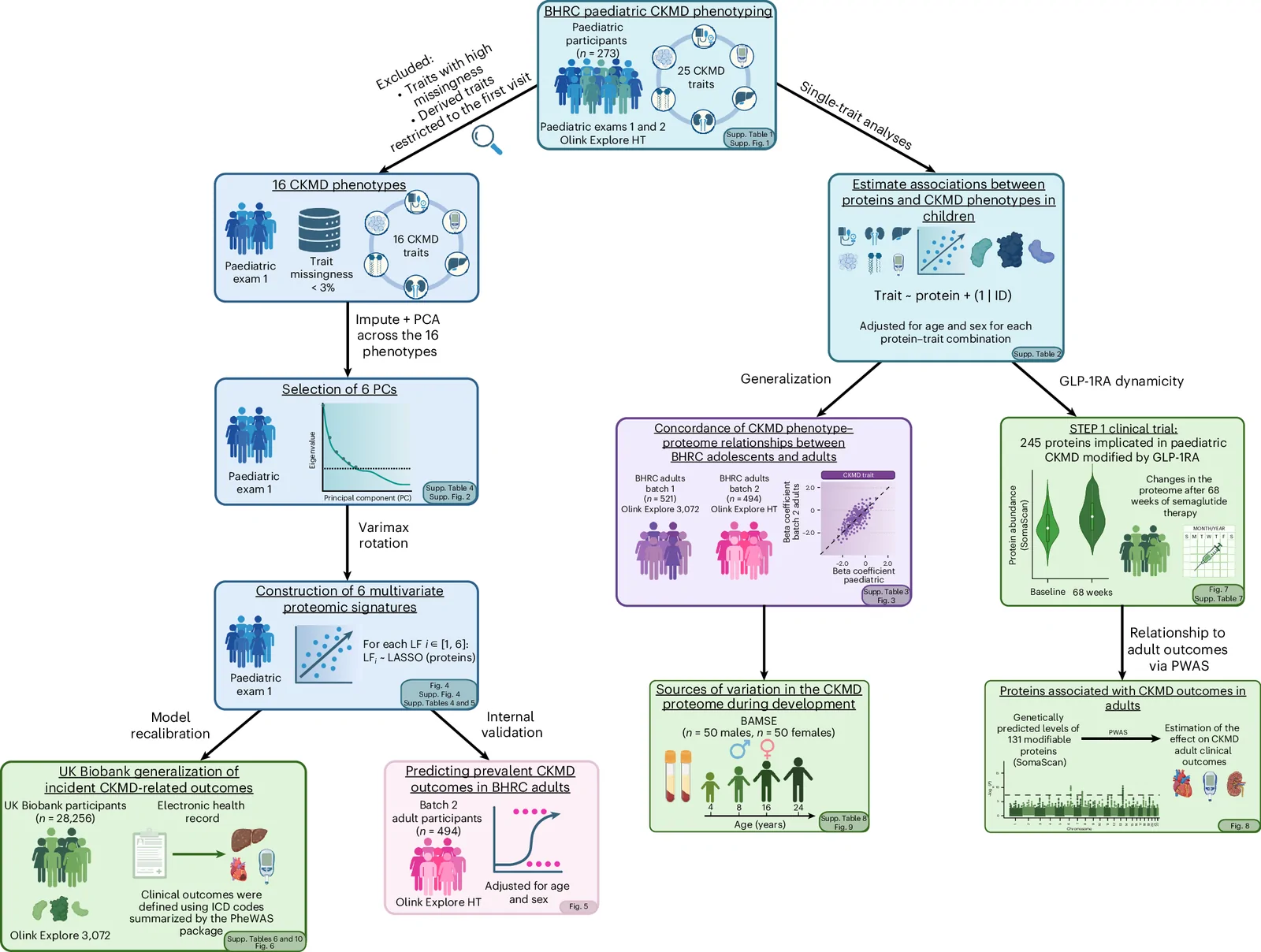
Study design. Flow of data and analytical methods in the study. Figure created in BioRender; Frankel, E. https://biorender.com/aqf2uk2 (2026).

**Extended Data Fig. 1:**
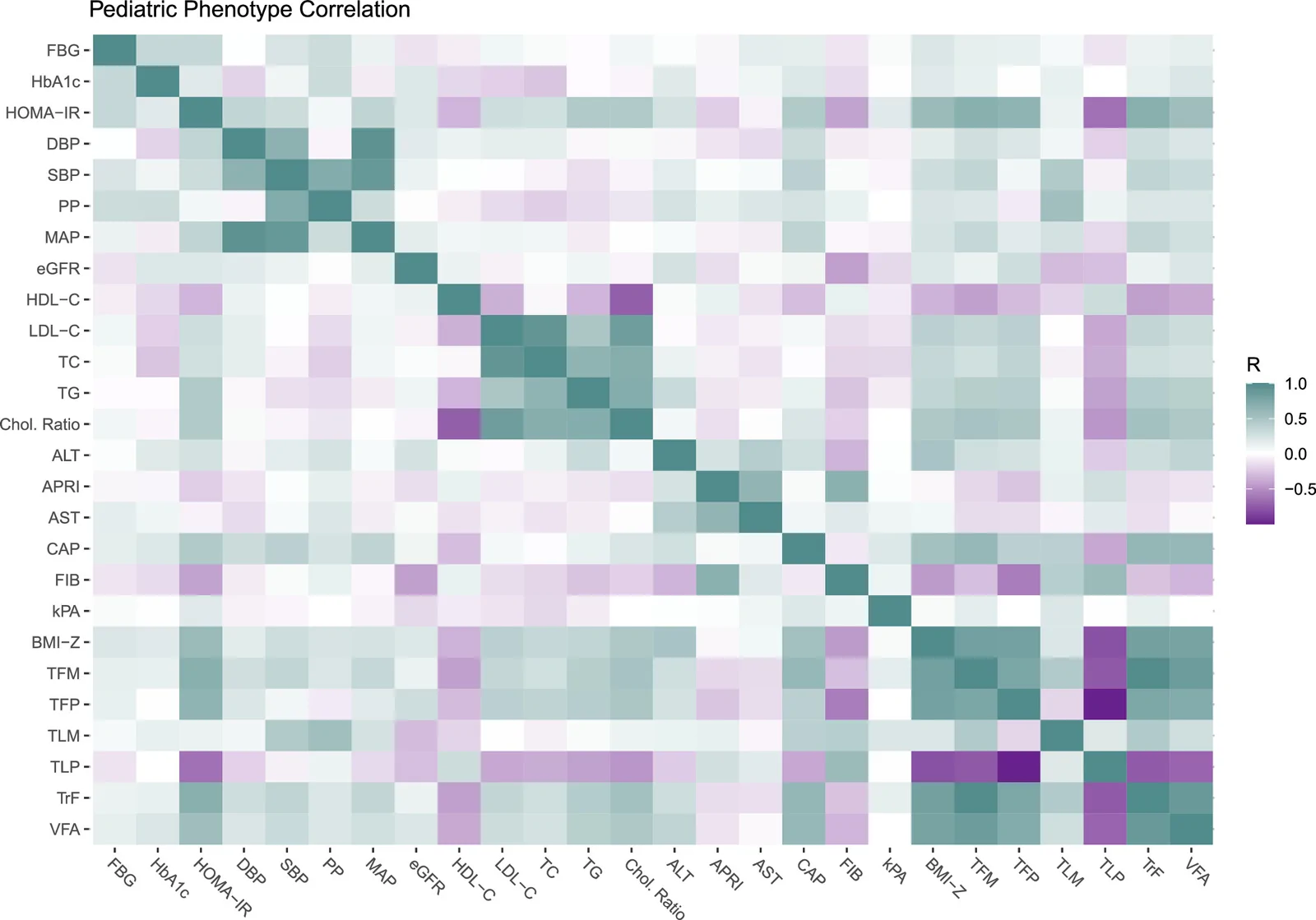
Phenotype correlation heatmap. This heatmap shows the correlations between each pair of measured phenotypes in the pediatric study sample. Full data is available in Supplementary Table 11. Source data

Paediatric participants were predominantly adolescents (13.1 ± 2.7 years of age), with approximately 53% self-reporting female sex (Table 1). More than a third of the Border Health Research Cohort (BHRC) paediatric participants had obesity with indications of pro-atherogenic dyslipidaemia (high triglycerides (TG) and low high-density lipoprotein (HDL-C)), hypertension and insulin resistance. Adult participants from the same cohort, distinct from the paediatric participants but including some first-degree relatives, were at similarly high risk for these conditions (51.5 ± 14.3 years of age, 54.1% with obesity, 39.8% with T2D, 68.3% females). We observed expected associations among CKMD phenotypes in youth, with body composition associated with greater insulin resistance, body fat distribution, pro-atherogenic dyslipidaemia and elevated blood pressure (Extended Data Fig. 1). The overall analytical flow for all results is shown in Fig. 1.

**Table 1:** Demographic summary of BHRC children and adults in proteomics datasets

Domain	Trait (units)	Abbreviation	Multi-trait analysis	Paediatric mean (s.d.)	Paediatric *n*_ind_	Adult mean (s.d.)	Adult *n*_ind_
	Age (years)		No	13.1 (2.7)	273	51.5 (14.3)	494
	Sex, *n* (% female)		No	145 (53%)	273	356 (68.3%)	494
Dysglycaemia	Fasting blood glucose (mg dl^−1^)	FBG	Yes	88.63 (6.97)	270	92.2 (8.8)	289
	HbA1c (%)	HbA1c	Yes	5.34 (0.29)	271	5.54 (0.32)	245
	HOMA-IR	HOMA-IR	Yes	3.1 (1.9)	260	2.4 (1.6)	259
Blood pressure	Diastolic blood pressure (mm Hg)	DBP	No	62.2 (7)	273	73.8 (9.1)	486
	Systolic blood pressure (mm Hg)	SBP	Yes	102.8 (10)	273	122.9 (19.2)	486
	Pulse pressure (mm Hg)	PP	No	40.7 (6.7)	273	48.8 (14.4)	486
	Mean arterial pressure (mm Hg)	MAP	No	75.7 (7.5)	273	89.7 (11.5)	486
Kidney	eGFR (sex- and age-dependent equation)	eGFR	Yes	108.7 (18.6)	270	98.7 (18.7)	465
Dyslipidaemia	High-density lipoprotein (mg dl^−1^)	HDL-C	Yes	48.4 (10.7)	271	50.1 (14.5)	491
	Low-density lipoprotein (mg dl^−1^)	LDL-C	Yes	78.2 (22.5)	271	105.2 (32.5)	484
	Total cholesterol (mg dl^−1^)	TC	Yes	146.0 (26.1)	271	183.3 (37.4)	491
	Triglycerides (mg dl^−1^)	TG	Yes	97.4 (52.5)	271	145.3 (92.4)	491
Liver	Alanine aminotransferase (U l^−1^)	ALT	Yes	26.0 (21.0)	271	32.5 (18.3)	473
	Aspartate aminotransferase to platelet ratio index	APRI	Yes	0.24 (0.14)	271	0.32 (0.22)	464
	Aspartate aminotransferase (U l^−1^)	AST	Yes	21.4 (10.7)	271	24.4 (13.2)	477
	Controlled attenuation parameter (dB m^−1^)	CAP	No	245.8 (57.8)	148	288.1 (63.2)	437
	Fibrosis-4	FIB	Yes	0.23 (0.1)	271	0.97 (0.56)	462
	Liver stiffness measurement (kPa)	kPa	No	4.46 (1.57)	162	5.96 (4.67)	429
Adiposity	BMI *z*-score	BMI-Z	Yes	0.96 (1.18)	273	NA	NA
	BMI (kg m^−^^2^)		No	NA	NA	32.8 (8.9)	494
	Total fat mass (g)	TFM	No	21,563 (10,670)	148	32,161 (11,391)	356
	Total percentage fat	TPF	No	36.0 (9.3)	148	41.21 (8.98)	356
	Total lean mass excluding bone mass (g)	TLM	No	34,712 (10,555)	148	42,942 (10,577)	356
	Total percentage lean excluding bone mass	TPL	No	60.8 (8.9)	148	55.97 (8.66)	356
	Trunk fat (g)	TrF	No	9,475 (5,340)	148	15,964 (5,768)	356
	Visceral fat area (cm^2^)	VFA	No	73 (38)	148	199 (80)	356
Clinical classifications	Hypertension (AAP 2017 paediatric hypertension guidelines) (%)		No	7 (2.6%)	273	NA	NA
	Hypertension (SBP ≥ 130 mm Hg, DBP ≥ 80 mm Hg or taking antihypertensive medications) (%)		No	2 (0.70%)	273	232 (45.3%)	494
	Low HDL (<50 mg dl^−1^ females, <40 mg dl^−1^ males) (%)		No	105 (38.5%)	273	218 (44.4%)	494
	High triglycerides (>150 mg dl^−1^ or on lipid-lowering medication) (%)		No	35 (12.8%)	273	248 (50.4%)	494
	Obesity (BMI percentile > 95^th^% children, BMI > 30 kg m^−^^2^ adults) (%)		No	95 (34.8%)	273	269 (54.1%)	494
	Diabetes (diagnosed T2D, on diabetes medication, FBG > 126 mg dl^−1^ or HbA1c ≥ 6.5%) (%)		No	1 (0.4%)	273	207 (39.8%)	494

Traits analysed and clinical categorizations are listed as either mean (s.d.) or *n* (%). *n*_ind_, number of distinct individuals; NA, not applicable; 95th%, 95th percentile.

**Fig. 2:**
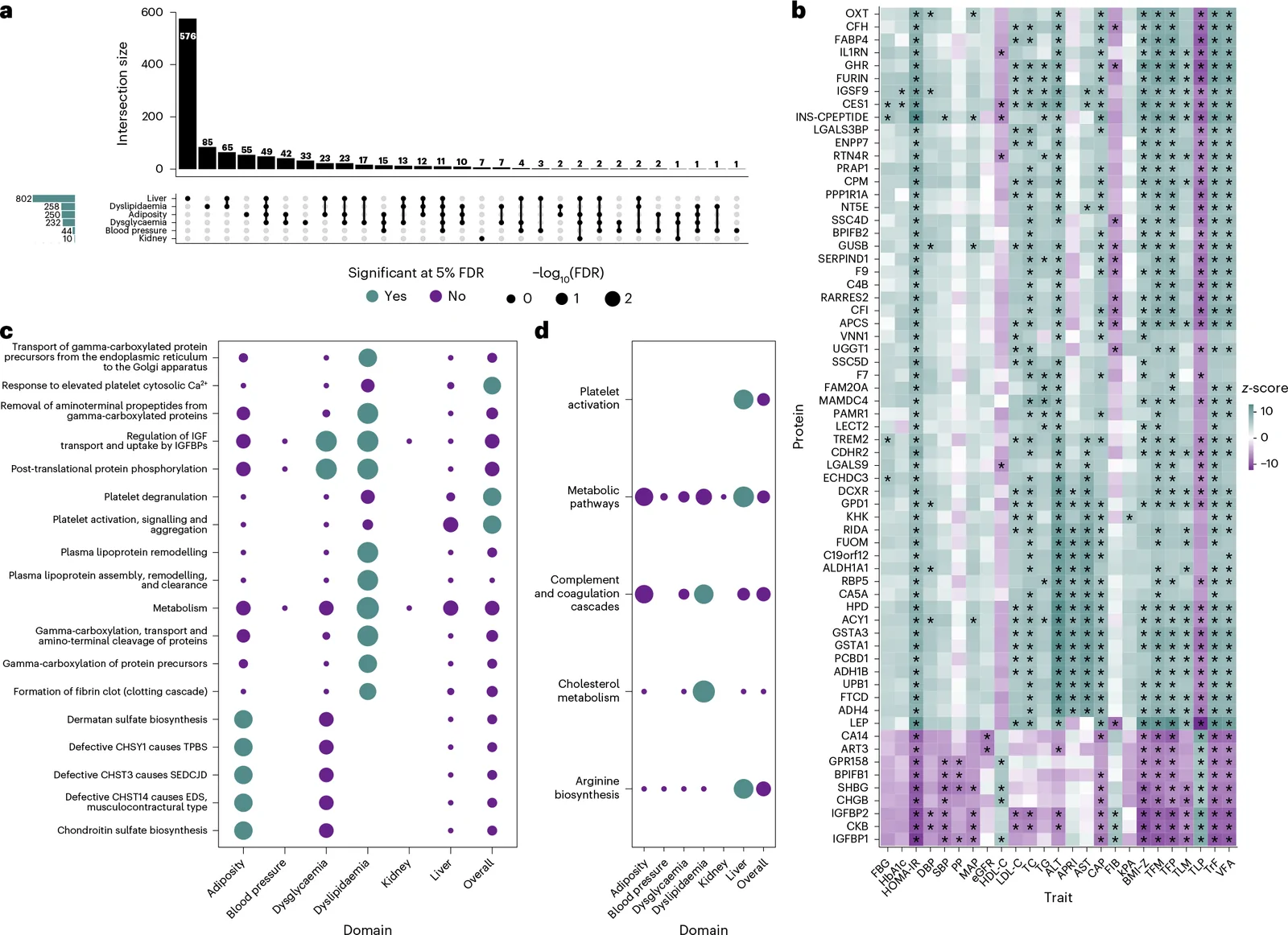
Proteomic architecture of CKMD in children. **a**, Significant proteins across 6 domains (all at the 5% FDR), showing shared association. **b**, Regression *z*-scores in linear mixed models (adjusted for age and sex) for proteins significant in at least four of the six phenotype domains. The asterisk denotes significance at 5% FDR. **c**,**d**, Reactome (**c**) and KEGG (**d**) pathway analyses. Full data for single-trait paediatric protein associations (**a** and **b**) are available in Supplementary Table 2a. Full data for **c** and **d** are available in Supplementary Table 9. CHSY1, chondroitin sulfate synthase 1; TPBS, Temtamy preaxial brachydactyly syndrome; CHST3, carbohydrate sulfotransferase 3; SEDCJD, spondyloepiphyseal dysplasia with congenital joint dislocations; CHST14, carbohydrate sulfotransferase 14; EDS, Ehlers–Danlos syndromes (EDS). Source data

We estimated the associations between the circulating proteome and the 25 paediatric CKMD phenotypes across the 6 defined domains (Supplementary Table 1), finding that 1,064 of the 5,420 unique proteins in our platform were associated with at least 1 phenotype. We identified 2,916 protein–phenotype associations (*q*-value < 0.05; the ‘CKMD proteome’; the full results are in Supplementary Table 2). A substantial fraction of the 1,064 associated proteins was statistically significant across multiple paediatric CKMD phenotypes (Fig. 2a), and 65 proteins were consistently associated with at least 4 CKMD domains (Fig. 2b). While a complete mechanistic ontology lies beyond the scope of this work, the paediatric CKMD proteome (see curated entries in Table 2) reveals coherent signals that align with key metabolic processes in CKMD.

**Table 2:** Selected functional characterization of proteins associated with multiple domains

Protein	Domains associated with protein in paediatric cohort	Direction	Putative function and previous studies
C-peptide	**Dysglycaemia**, blood pressure, liver, adiposity	+	Marker of beta-cell function and reserve, with higher levels suggesting hyperinsulinaemia; increased in children with impaired glucose tolerance after glycaemic challenge^10^
Immunoglobulin superfamily member 9 (IGSF9)	Dysglycaemia, blood pressure, dyslipidaemia, **liver**, adiposity	+	IGSF9 involved in cell adhesion; related to hepatic steatosis and fibrosis in adults with HIV^89^
Glycerol-3-phosphate dehydrogenase 1 (GPD1)	Dysglycaemia, blood pressure, dyslipidaemia, **liver**, adiposity	+	GPD1, involved in carbohydrate metabolism in the liver; mixed mechanistic findings, with congenital deficiency linked to hypertriglyceridaemia^90^ and liver disease^91^, while expression increased in adipose tissue in adults with obesity and is associated with BMI^24,25^; expression responsive to weight loss intervention^92^
Aminoacylase 1 (ACY1)	Dysglycaemia, blood pressure, dyslipidaemia, **liver**, adiposity	+	ACY1; high liver expression, levels in adults related to diabetes and hepatic steatosis^23,27,28^
Chromogranin B (CHGB)	**Dysglycaemia**, blood pressure, dyslipidaemia, liver, adiposity	−	CHGB; involved in secretion of catecholamines (nervous system) and in insulin granule trafficking^18^ (pancreatic beta-cells); genetic studies of *CHGB* variation and *Chgb*-null mice suggest lower levels related to higher blood pressure^19^; previous association with metabolic traits in adults^20^
Aldehyde dehydrogenase 1 family member A1 (ALDH1A1)	Liver	+	Aldehyde dehydrogenase; important in metabolic processing of aldehydes in the liver; expression in paediatric livers lower than adults^93^; increased expression may protect against hepatic oxidative stress and inflammation during liver injury^26^
Creatine kinase B (CKB)	Dysglycaemia, blood pressure, dyslipidaemia, liver, **adiposity**	−	CKB; involved in high-energy phosphate transfer in highly metabolic tissues; increase in endoplasmic reticular stress during obesity epigenetically reduces *CKB* expression, increasing inflammatory chemokine expression in fat^29^; reduction in CKB in mice impairs thermogenesis and increases obesity^30^
Beta-glucuronidase (GUSB)	**Dysglycaemia**, blood pressure, dyslipidaemia, liver, adiposity	+	GUSB; previous studies in adults link circulating levels to diabetes^21^
Insulin-like growth-factor binding protein (IGFBP1 and IGFBP2)	**Dysglycaemia**, blood pressure, dyslipidaemia, liver, **adiposity**	−	Insulin-like growth-factor binding proteins; linked to lower risk of hepatic steatosis, obesity, atherosclerotic heart disease and insulin resistance^22,23^; previous evidence in children suggests IGFBP1 is an early marker of insulin resistance^31^
Sex hormone-binding globulin (SHBG)	**Dysglycaemia**, blood pressure, dyslipidaemia, **liver**, adiposity	−	SHBG; involved in sex hormone metabolism; early reduction in SHBG related to faster decline in insulin sensitivity independently of weight^32^; levels are lower in children with obesity, reversible with weight loss, with higher levels linked to hepatic steatosis and widespread metabolic dysfunction^33^
β-site amyloid precursor protein cleaving enzyme 1 (BACE-1)	**Dysglycaemia**, adiposity	−	Overexpression can lead to increased hepatic insulin resistance in murine models, potentially via BACE-1-mediated cleavage of the insulin receptor; pharmacologic targeting of BACE-1 can abrogate these effects^37,38^
G-protein coupled receptor 158 (GPR158)	**Dysglycaemia**, blood pressure, dyslipidaemia, **adiposity**	−	Involved in CNS-based regulation of energy metabolism and nutrient intake^39^; variants in GPR158 may impact liability to weight gain^40^
Guanylate binding protein 1 (GBP1)	**Liver**	+	Involved in pyroptosis in inflammatory liver injury^94^
Fibroblast growth-factor binding protein 3 (FGFBP3)	**Dyslipidaemia**	+	FGFBP3-null mice have lower lipid and TG levels, and supplementation of FGFBP3 in mice with obesity can ameliorate metabolic dysfunction, weight gain and liver steatosis; mechanism via interaction with FGFs^41^
Ubiquitin specific peptidase 28 (USP28)	**Liver**	+	High expression in human metabolic-associated steatotic liver disease and steatohepatitis; functions via stabilizing PPAR-gamma via blocking ubiquination; lack of USP28 may lead to reduction in PPAR-gamma signalling and reduced hepatic inflammation^42^
Cysteine-rich with EGF-like domains 2 (CRELD2)	**Liver**	−	May limit ER stress and resilience; lack of CRELD2 may augment unfolded protein response and contribute to MASLD and changes in energy expenditure; may even have sex-specific effects^43^
Fibroblast growth-factor receptor 4 (FGFR4)	**Dysglycaemia**, adiposity	−	Divergent results; FGFR4 modulates insulin sensitivity in murine obesity models^95,96^
Cathepsin B (CTSB)	Dysglycaemia, **adiposity**	−	Lysosomal protease; increased in white adipose tissue in mice; may regulate perilipin-1, which regulates lipolysis in fat^97^
Transmembrane protein 108 (TMEM108)	**Adiposity**	−	Traditionally implicated in psychiatric disorders; TMEM108 mutant mice have poorer insulin sensitivity and shift metabolism towards glucose (away from lipid); metformin can rescue this effect^98^

This list includes proteins related to five domains in the BHRC paediatric cohort (ACY1, ALDH1A1, CHGB, CKB, GPD1, GUSB, IGFBP1, IGFBP2, IGSF9, C-peptide and SHBG) as well as additional selected proteins related to one or more domains with previous evidence in CKMD. Domains in bold reflect those with previous functional evidence (previous studies provided).

+, higher protein abundance is more adverse; −, lower protein abundance is more adverse. CNS, central nervous system; ER, endoplasmic reticular; FGFs, fibroblast growth factors; PPAR, Peroxisome proliferator-activated receptor.

### Characterizing the paediatric CKMD proteome

Among proteins associated with glycaemic control, we observed markers indexing distinct facets of β-cell function and insulin sensitivity. For example, C-peptide^10^ (an indicator of residual β-cell secretory reserve) was associated with many metabolic measures, reinforcing the value of measuring endogenous insulin secretion in early disease. Meanwhile, chromogranin B (CHGB), known to modulate insulin granule trafficking in β-cells by facilitating proinsulin transit from the trans-Golgi to the plasma membrane, was inversely related to dysglycaemia in our paediatric cohort, echoing adult findings of CHGB influence on metabolic traits^18–20^. In addition, canonical adult markers of insulin sensitivity, such as GUSB^21^, insulin-like growth-factor binding protein 1 (IGFBP1) and IGFBP2, were detected and have shown consistent directionality in children^22,23^. For hepatic traits, significant proteins implicate core metabolic machinery in the liver. For example, glycerol-3-phosphate dehydrogenase (GPD1) has a central role in glycerolipid metabolism and gluconeogenesis^24,25^, while aldehyde dehydrogenase 1 family member A1 (ALDH1A1) is a key node in retinoid and aldehyde detoxification pathways^26^. Some proteins, such as immunoglobulin superfamily member 9 (IGSF9), have been previously linked in adult cohorts to steatosis or fibrosis. Aminoacylase 1 (ACY1) has been identified in previous metabolic disease or hepatic injury studies^23,27,28^.

**Extended Data Fig. 2:**
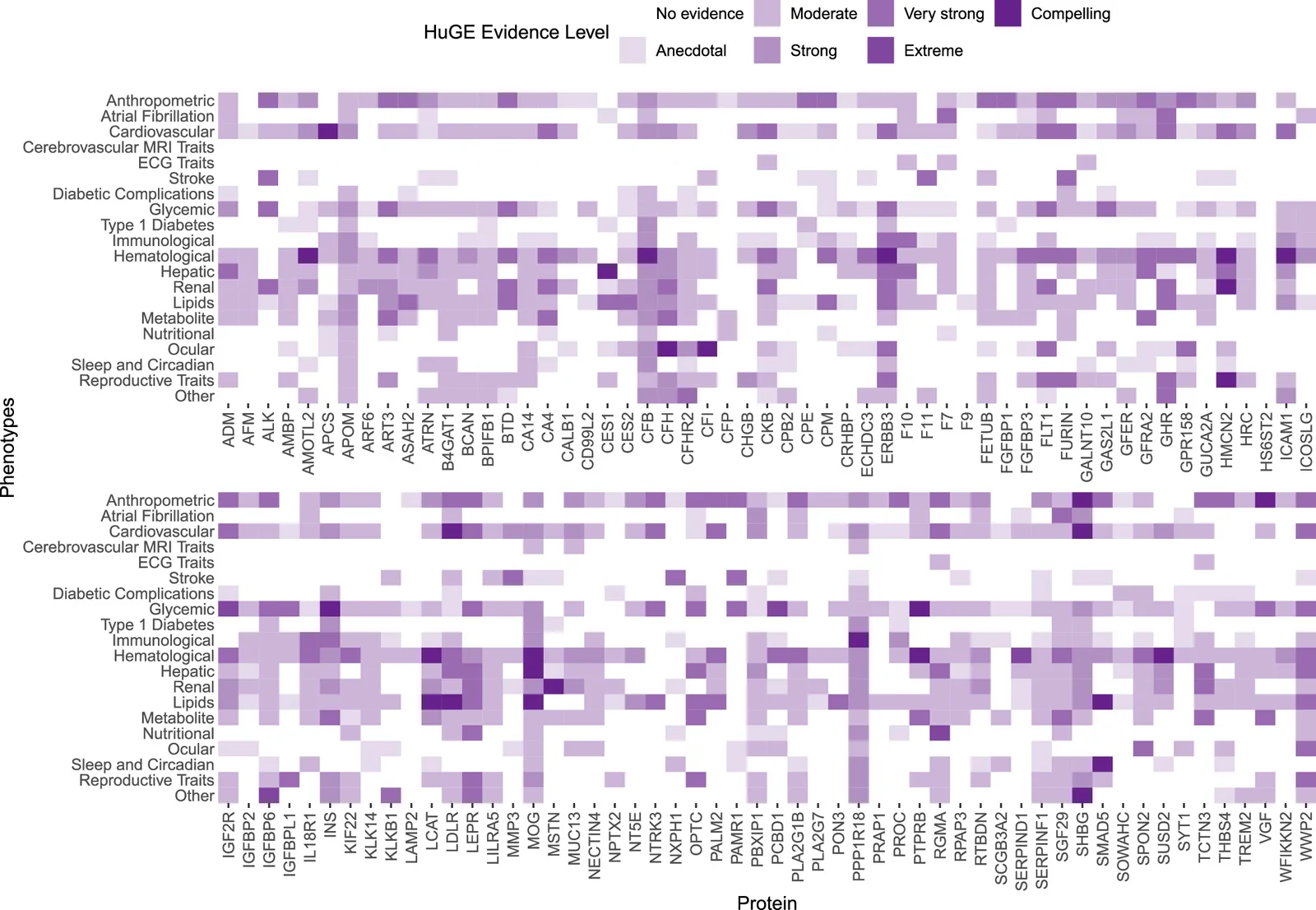
HuGE genetic evidence heat map. Supplementary Fig. 2 Genetic evidence (HuGE) for select proteins from pediatric studies (as described in Methods). Source data

Our results span both protective and risk-conferring associations with the CKMD phenome. For instance, elevated creatine kinase B (CKB), a key enzyme in phosphagen energy shuttling that has been implicated in thermogenesis in highly metabolic tissues, was associated with adiposity-related phenotypes in children. This is consistent with murine knockout studies where CKB influences energy expenditure, inflammation in adipose tissue and obesity outcomes^29,30^. Similarly, CHGB’s association with lower insulin resistance fits with its secretory role and with adult metabolic directionality^19^. We also confirmed previous findings in paediatric populations: IGFBP1 and IGFBP2 (markers of insulin sensitivity) and sex hormone-binding globulin (SHBG), for which rapid early declines predict insulin resistance independently of adiposity, all aligned with our proteomic signals^31–33^. In addition, proteins long implicated in adult CKMD—such as leptin (LEP), fatty acid-binding protein 4 (FABP4) and retinoic acid receptor responder 2 (RARRES2)—were recapitulated in children, suggesting potential similarities in molecular processes across the lifespan^34–36^. In addition to well-established proteins, our analysis also revealed several proteins with plausible mechanistic roles but limited previous evidence in CKMD. These include β-site amyloid precursor protein cleaving enzyme 1 (BACE-1), G-protein coupled receptor 158 (GPR158), fibroblast growth-factor binding protein 3 (FGFBP3), ubiquitin specific peptidase 28 (USP28) and cysteine-rich with EGF-like domains 2 (CRELD2), among others^37–43^. Such proteins operate in signalling, cellular stress or turnover pathways, as further detailed in Table 2, and represent candidates for future functional follow-up. Pathway analysis across the CKMD proteome further revealed associations with pathways of cholesterol metabolism, inflammation and haemostatic mechanisms, and metabolism (Fig. 2c,d). Indeed, mapping to the Human Genetic Evidence (HuGE) framework suggests external genetic support for many of the targets in the paediatric CKMD proteome with CKMD traits studied in adults, including anthropometric, cardiovascular, hepatic and renal phenotypes (Extended Data Fig. 2). Pairwise correlations across proteins indicated some correlation across proteins (average Spearman correlation, 0.258; Supplementary Fig. 1).

### Similarities between the paediatric and adult CKMD proteome

**Fig. 3:**
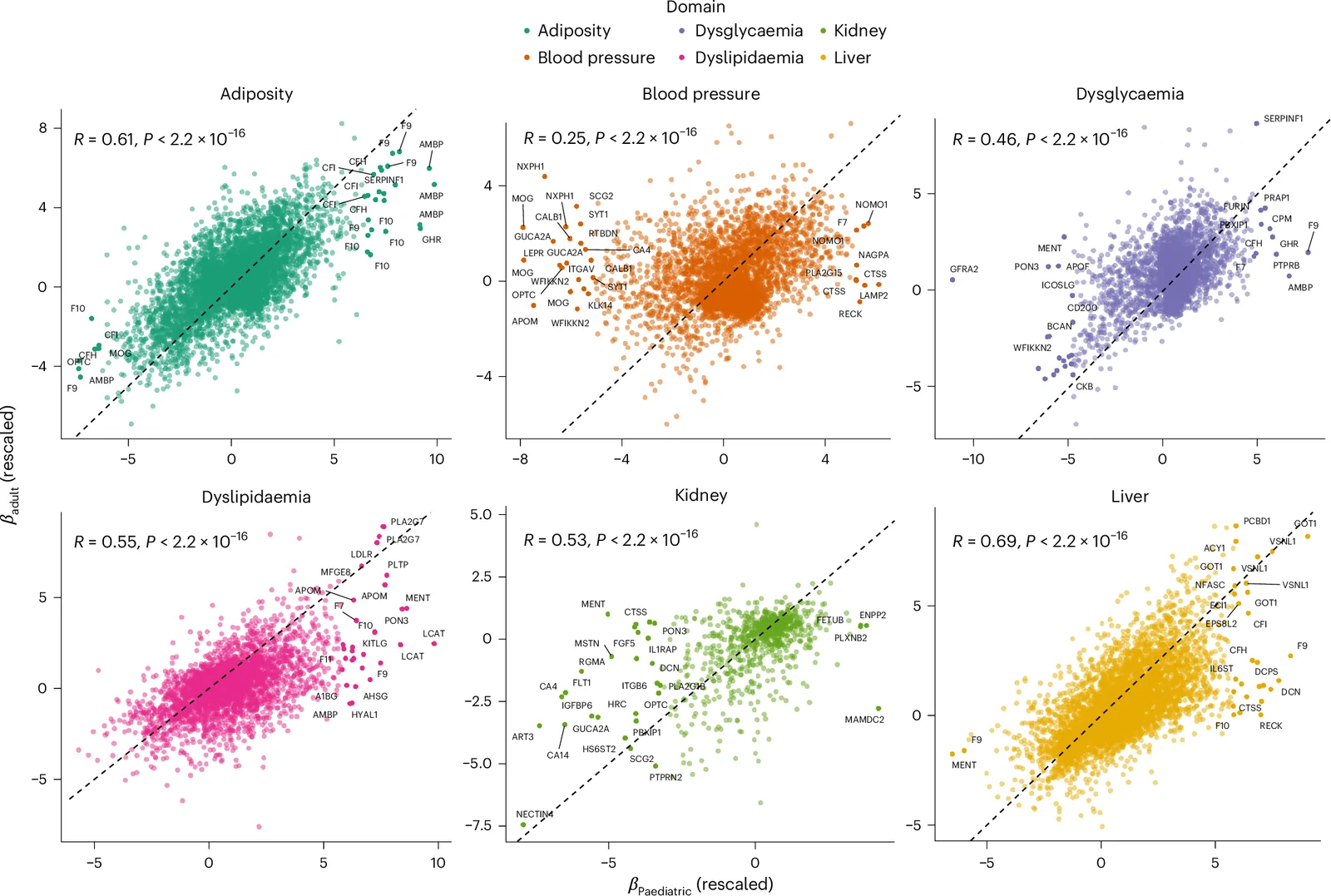
Concordance of CKMD phenome–proteome relationships between adults and youth. This figure depicts the correlation of paediatric and adult regression model effect sizes. The *x* axis shows rescaled beta coefficients for paediatric results, and the *y* axis shows rescaled beta coefficients for adult batch 2 results. Protein labelling is arbitrary. Each protein–trait association represents a distinct single-trait model. Pearson correlations were two-sided and not corrected for multiple testing. The full data are available in Supplementary Tables 2a and 3. Source data

Understanding links between the serum proteome and CKMD phenotypes in children—specifically, whether these links recapitulate relationships observed in adults—is key in identifying nascent, potentially modifiable features of CKMD. Across 685 adults in the BHRC and up to 25 phenotypes measured in both children and adults (Table 1), we observed a broad concordance in the magnitude and directionality of phenotype–protein relationships (Fig. 3 and Supplementary Table 3). Of the 2,916 significant paediatric protein–phenotype associations, 1,877 (64%) were significant and had directionally consistent effects. Across domains, we observed the strongest paediatric–adult correlation for the adiposity (*r* = 0.61) and liver domains (*r* = 0.69). While many proteins showed expected associations with phenotypic data (for example, ACY1, GOT1 with hepatic steatosis and fibrosis), we also observed associations with divergent directions in children and adults for some phenotypes, some of which have demonstrated pathophysiologic roles in CKMD (for example, PON3 (ref. ^44^), APOF^45^ for some dysglycaemia phenotypes) as well as those with no previous evidence in CKMD (for example, MENT, NXPH1).

### Generating a paediatric proteomic signature of CKMD

**Fig. 4:**
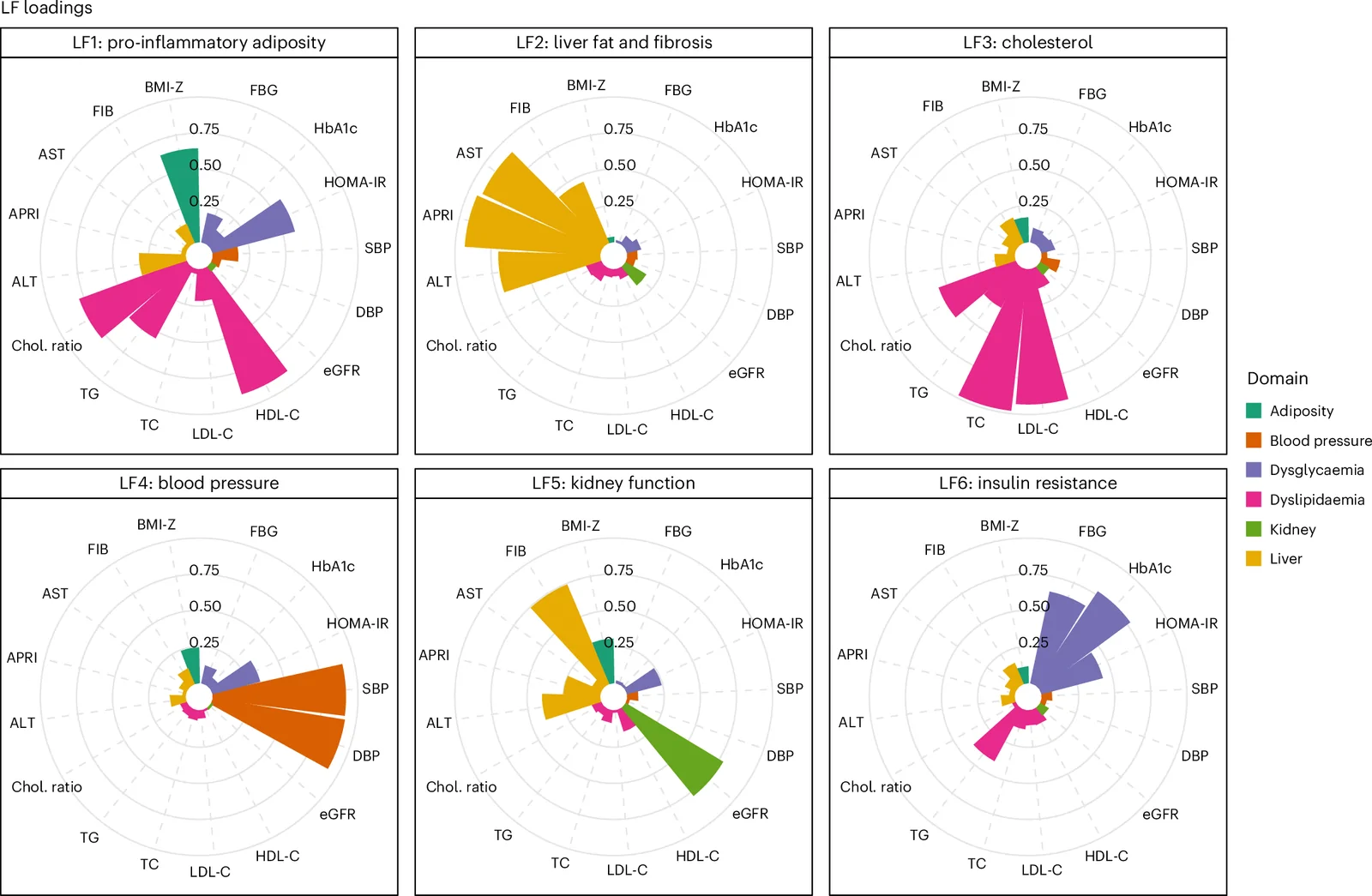
Varimax-rotated principal components (LFs) of the paediatric CKMD phenome. These circular bar plots indicate the magnitude of the loadings per trait for each LF of the paediatric phenotype matrix. The bars are coloured by the domain of each trait. Abbreviations are as in Table 1. Chol. ratio, ratio of TC to HDL-C. Source data

The biological plausibility and concordance of many proteome–CKMD phenome associations across adults and children are consistent with an early nascent phase of CKMD; still, identifying clear links to clinical risk factors or outcomes remains difficult, given the extended time horizon from childhood needed to capture clinical events. Therefore, we next constructed multi-protein-based signatures of CKMD phenotypes in children to test their relevance for clinical CKMD end-points in adults in multiple populations. We used principal components analysis (PCA) to summarize 16 phenotypes measured in both children and adults, retaining 6 varimax-rotated, orthogonal latent factors (LFs) explaining ≈77% of the overall variation in the measured paediatric phenome (Supplementary Fig. 2). For interpretability, we labelled LFs by clinical interpretation of features with high loadings, recognizing that each LF was loaded heterogeneously on CKMD phenotypes (Fig. 4 and loadings in Supplementary Table 4): (1) pro-inflammatory adiposity (LF1: primarily loaded on pro-atherogenic lipids, homeostatic model of insulin resistance (HOMA-IR) and body mass index (BMI Z score)); (2) liver fat and fibrosis (LF2: loaded on transaminases and aspartate aminotransferase to platelet ratio index (APRI)); (3) cholesterol (LF3: loaded on low-density lipoprotein (LDL) and total cholesterol (TC)); (4) blood pressure (LF4: loaded on systolic blood pressure (SBP) and diastolic blood pressure (DBP)); (5) kidney function (LF5: loaded on estimated glomerular filtration rate (eGFR) and liver fibrosis); and (6) insulin resistance (LF6: loaded on glycated haemoglobin (HbA1c), HOMA-IR and fasting glucose). The clinical interpretation of ‘higher’ LF values varied depending on loading directionality, with higher values for all LFs but LF3 representing a more adverse CKMD state. We generated proteomic signatures of each LF in least absolute shrinkage and selection operator (LASSO) regression to serve as molecular scores of the six summary traits. Our model coefficients are in Supplementary Table 5; the mean cross-validated *R*^2^ for each paediatric model and number of proteins per model, *k*, for each LF are as follows: LF1: *R*^2^ = 0.485, *k* = 154; LF2: *R*^2^ = 0.560, *k* = 89; LF3: *R*^2^ = 0.256, *k* = 70; LF4: *R*^2^ = 0.072, *k* = 20; LF5: *R*^2^ = 0.359, *k* = 52; and LF6: *R*^2^ = 0.142, *k* = 63. Coefficients for the top 50 model coefficients by feature importance (or fewer, for smaller models) for each LF-based model are shown in Supplementary Fig. 3.

### Paediatric CKMD proteomic signatures identify adults with susceptibility to clinical CKMD

**Fig. 5:**
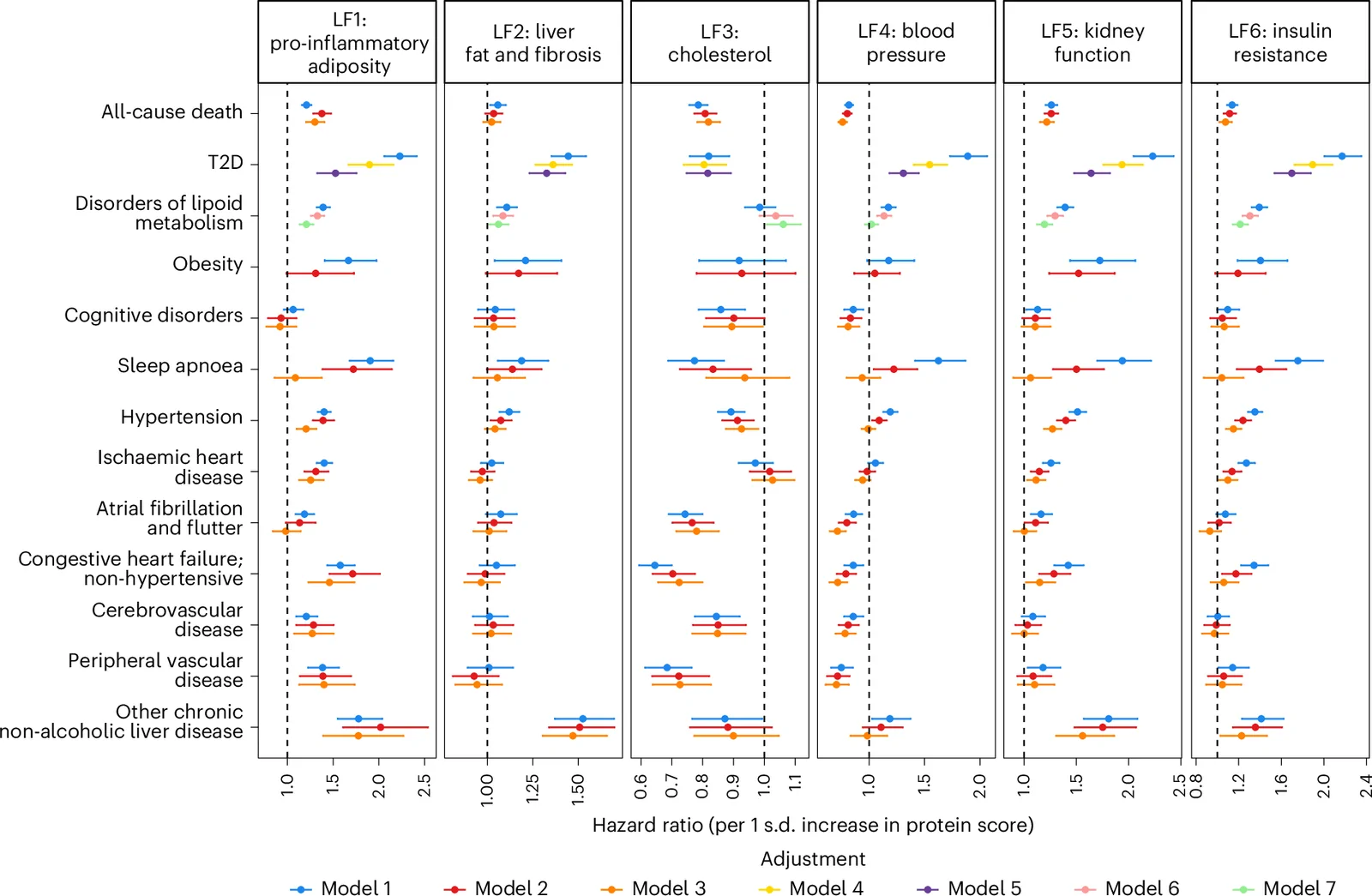
Long-term implication of the paediatric CKMD proteome on adult clinical risk. The forest plot depicts the hazard ratio (Cox models) of childhood-derived proteomic scores for CKMD-related end-points in the UK Biobank. The colour of the marks in the plot indicates for which variables each model was adjusted. The error bars represent 95% confidence intervals of the hazard ratio. The models control for covariates as follows: model 1: age, age^2^, sex, ethnicity, smoking, alcohol and Townsend deprivation index; model 2: model 1 + HbA1c + TG + HDL; model 3: model 2 + BMI; model 4: model 1 + TG + HDL; model 5: model 1 + BMI + TG + HDL; model 6: model 1 + HbA1c; and model 7: model 1 + BMI + HbA1c. Source data

**Extended Data Fig. 3:**
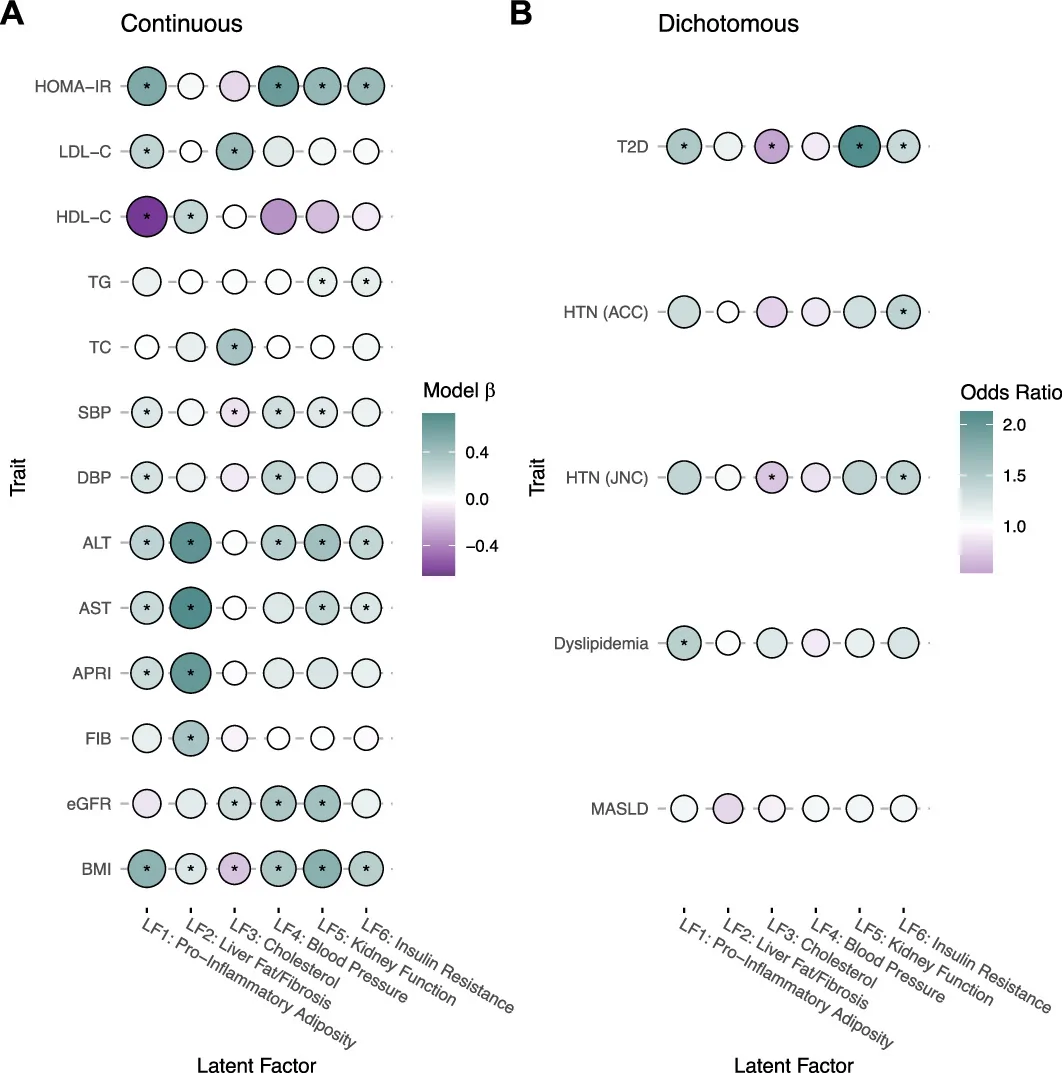
Mapping proteomic signatures of the pediatric CKMD proteome to adults. Age- and sex-adjusted association of each proteomic score (built on latent factors of the pediatric CKMD phenome) in up to 521 Hispanic/Latino adults from the same population. Size and color intensity represent the magnitude of the effect size. The * indicates significance at FDR-adjusted P 0.05. Correlations are Pearson. Panel (**a**) displays estimates for outcomes that are continuous and panel (**b**) displays estimates for outcomes that are dichotomous. Full data in Supplementary Table 10. Source data

We next examined whether proteomic signatures of paediatric CKMD would be associated with CKMD in adults. We first conducted a cross-sectional analysis in 494 Hispanic or Latino adults from the same community, applying the paediatric proteomic LF scores to adult proteomic data and testing associations with CKMD-associated measures. We found that proteomic signatures, derived in children and adolescents, were associated with analogous traits in corresponding LFs in adults, suggesting that paediatric proteomic signatures may be related to comparable CKMD profiles across paediatric and adult populations (Extended Data Fig. 3). Next, we estimated associations between these paediatric CKMD signatures and incident clinical CKMD in adults in the UK Biobank (characteristics in Extended Data Table 1; median follow-up 13.7 years, 25th–75th percentile 13.0–14.5 years for mortality). Pro-inflammatory adiposity (LF1), liver fat and fibrosis (LF2) and insulin resistance (LF6) (for which higher values in childhood signified more advanced paediatric CKMD) were associated with higher risk of all-cause mortality, T2D, multiple cardiovascular diseases, sleep apnoea and fatty liver disease (Fig. 5). Blood pressure (LF4) and kidney function (LF5)—also associated with more advanced paediatric CKMD—were linked to higher T2D risk, with heterogeneous associations with other conditions. By contrast, cholesterol (LF3), for which higher values in childhood were less adverse, had protective associations with cardiovascular and metabolic conditions in adults. Most of our observed associations were robust to comprehensive covariate adjustment in adults (Supplementary Table 6). Collectively, these findings underscore the potential consistency of early CKMD risk, as reflected in the childhood proteome, with the diagnosis of adult cardiovascular and metabolic disease.

### Modifiability of proteins associated with paediatric CKMD traits during GLP-1RA therapy in adults

**Fig. 6:**
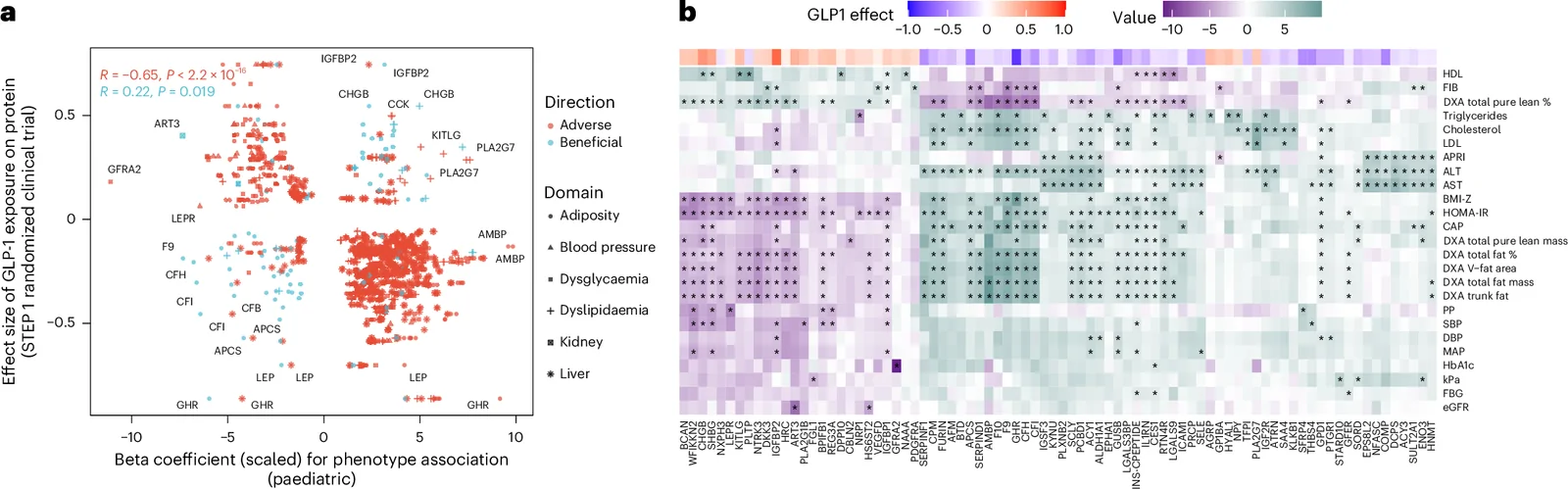
Dynamicity of the circulating paediatric CKMD proteome with GLP-1RA therapy in adults. **a**, Paediatric CKMD proteome across the 6 domains matched to proteomic data from the STEP 1 clinical trial, showing the changes in circulating proteins after 68 weeks of therapy. Effect sizes and *P* values were generated from linear regression models, and *P* values were corrected for multiple testing using the Holm–Bonferroni (STEP 1) and Benjamini–Yekutieli (BHRC) methods. For visualization, results with an FDR > 5% in either cohort (STEP 1 or BHRC) are not shown. Points are coloured based on the parent phenotype (adverse or beneficial based on the nature of the phenotype; for example, lean body mass is designated ‘beneficial’, whereas BMI is designated ‘adverse’). Generally, we observed that proteins with adverse relations to CKMD phenotypes were decreased with GLP-1RA therapy and proteins with beneficial relations were increased. **b**, Visualization of the top 10 proteins (ranked by effect size) related to individual CKMD phenotypes in the paediatric cohort (with several proteins overlapping among phenotypes). Proteins that are positively associated with phenotypes (for example, BMI, HOMA-IR) were generally reduced after GLP-1RA therapy. The full data are available in Supplementary Table 7. Pairwise protein correlations are included in Supplementary Fig. 1. Source data

Beyond statistical associations with CKMD, we expect the most clinically relevant biomarkers to change in response to disease-modifying therapy. To explore the potential modifiability of protein levels implicated in paediatric CKMD risk in our previous analyses, we mapped 1,032 proteins associated with any CKMD outcome to data from adult participants in the STEP 1 clinical trials of semaglutide (Methods)^15^. Protein levels associated with more adverse CKMD in children and adolescents were altered in a favourable direction following semaglutide therapy in the STEP 1 sample (Fisher’s exact test *P* < 2.2 × 10^−16^; Fig. 6a and Supplementary Table 7). These findings may be mediated through a variety of systemic changes, including weight loss. Notable examples included LEP, FABP4, CES1 and ACY1, which are all associated with greater paediatric insulin resistance, adiposity and liver fat and fibrosis, and having plausible CKMD-relevant mechanisms in adults (all reduced among adults on GLP-1RA). Conversely, SHBG, WFIKKN2 (ref. ^27^) and IGFBP2 represent examples of proteins with generally opposing effects relative to LEP in the paediatric sample and increased after GLP-1RA treatment in adults. We also identified proteins associated with early CKMD in our paediatric cohort (for example, BCAN, ART3) that have not previously been implicated in adult T2D or CKMD. A heat map of the top 10 proteins (ranked by effect size) for each phenotype in the paediatric cohort is shown in Fig. 6b. Proteins implicated by the CKMD phenotypes in children that showed evidence of changes in concentration with GLP-1RA therapy in adults appeared distributed across key tissues and cell types, with strong representation of hepatocytes, pancreatic cells, adipose tissue and brain tissue (Supplementary Fig. 4). While we cannot disentangle the relative contributions of GLP-1RA-mediated weight loss versus other metabolic effects on individual CKMD-related proteins, these findings suggest that many proteomic signatures associated with paediatric CKMD risk are favourably modifiable with modern CKMD-targeted therapy in adults.

### Genetic liability of the dynamic paediatric CKMD proteome is linked to lifetime CKMD

We next studied whether genetic liability of the CKMD proteome is linked to key CKMD phenotypes in adults, a window into lifelong CKMD susceptibility associated with a proteome identified in childhood. We performed a PWAS using large published genome-wide association studies (GWAS) of heart failure, BMI, T2D, renal function, myocardial infarction and metabolic-associated steatotic liver disease (MASLD) spanning up to 2.5 million individuals in a single trait (see Methods for sample sizes). This approach is aimed at using genetic models of circulating proteins linked to CKMD in childhood to quantify the relation with adult CKMD outcomes. To restrict our analyses to proteins potentially dynamic and linked to CKMD, we limited our PWAS analysis to 131 proteins that (1) were associated with paediatric CKMD phenotypes (*q*-value < 0.05), (2) had evidence of changing after GLP-1RA therapy in adults (*q*-value < 0.05) and (3) had models of genetically predicted expression.

**Extended Data Fig. 4:**
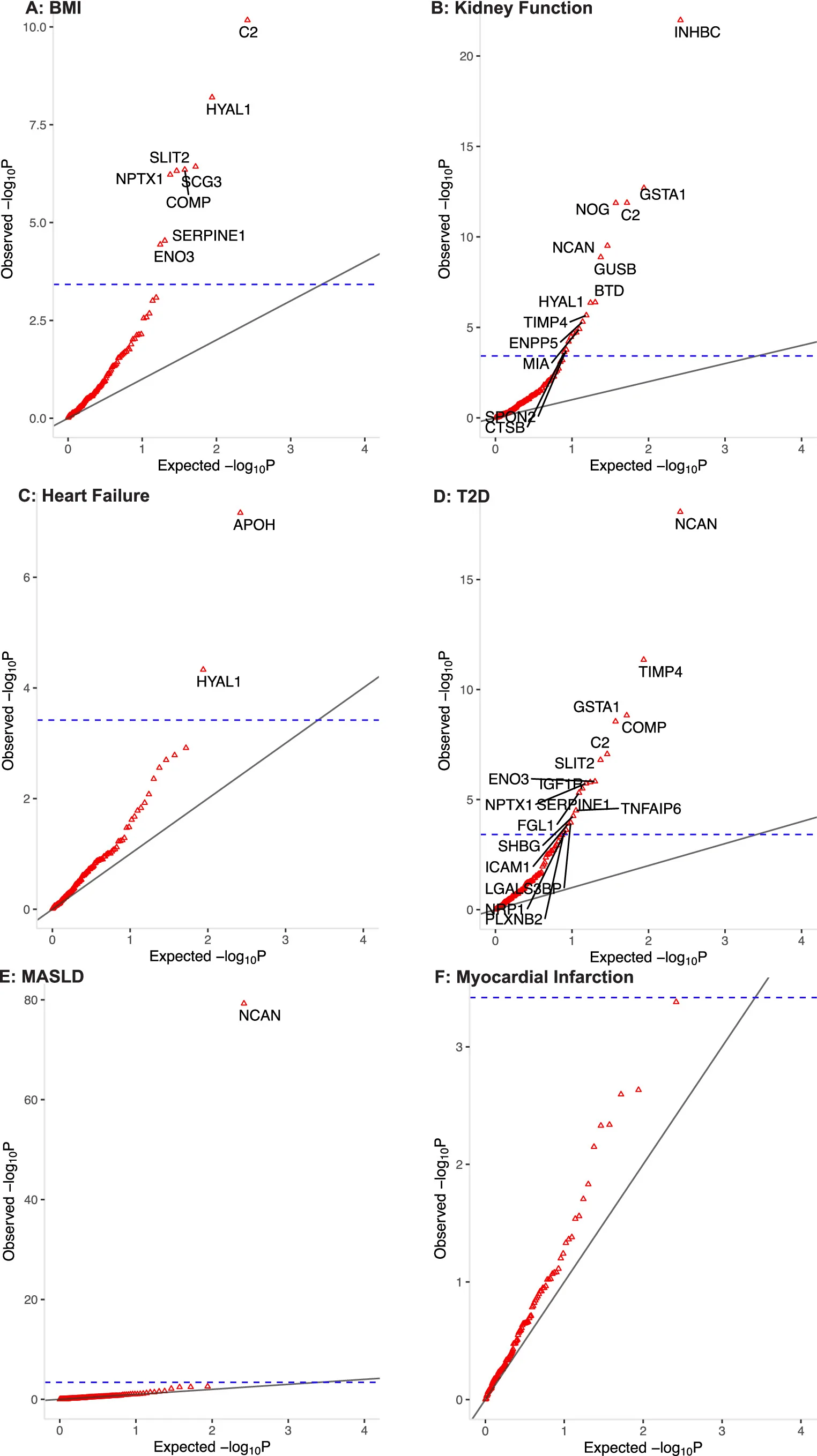
Proteome-wide association studies (PWAS) for CKMD outcomes in adults. Each panel represents the results of PWAS for proteins that were (1) associated with CKMD phenotypes in BHRC children, (2) responsive to GLP-1RA therapy in adults, and (3) had models of genetically predicted expression. The diagonal line represents complete agreement with the null hypothesis; an upward/leftward shift from the diagonal line represents an enrichment of significant associations compared to what was expected under the null. The blue dotted line represents a Bonferroni p-value threshold of 0.05 across the proteins tested. Proteins above this line (labeled) exhibit genetic evidence for association with the target outcome. Panels **a**–**f** detail associations for each outcome: BMI, kidney function, heart failure, T2D, MASLD, and myocardial infarction. Source data

We observed strong associations with multiple CKMD outcomes (Extended Data Fig. 4), including targets with cross-system and canonical physiologic roles with potential therapeutic relevance. We observed paediatric CKMD targets that reflect BMI-specified mechanisms of energy homeostasis (for example, SLIT2, previously implicated in beige fat thermogenesis improvement of glucose sensitivity^46^), appetite (for example, SCG3, previously implicated in food intake regulation^47^), and adipose tissue inflammation and adipogenesis (SERPINE1/PAI-1 (ref. ^48^), COMP^49^). Paediatric CKMD-related proteins also implicated pathways across multiple CKMD mechanisms and outcomes, including activin and TGF-beta signalling (INHBC^50,51^), matrix remodelling (TIMP4), oxidative homeostasis (GSTA1), autophagy (SPON2 (ref. ^52^)) and lipoprotein metabolism (APOH, implicated in lipoprotein(a) metabolism^53^). Of note, NCAN was strongly related to both MASLD and T2D, a finding supported by previous studies in MASLD in adult populations^54^ but not widely in T2D. In addition to known targets, this proteogenomic approach also identified targets not previously widely reported (for example, hyaluronan catabolism with potential roles in T2D and CKMD^55^). Collectively, these results provide genetic support for a consistent set of CKMD-related proteins identified by discovery in a paediatric population and prognostic for CKMD outcomes in adults.

### Early developmental variation in a shared paediatric–adult CKMD proteome

The variability of CKMD-linked proteins during the adolescent–adulthood transition, and whether they reflect developmental age- or sex-related changes, are critical to positioning their use as potential biomarkers of early CKMD. To address this, we leveraged a recently reported longitudinal study of 100 individuals from age 4 to 24 years in the Swedish BAMSE cohort^17^ to examine sources of variability in protein abundance. Focusing on proteins with evidence of significance (*q*-value < 0.05) in children that replicated in BHRC adults, we observed broad variability in the CKMD-associated proteome (Supplementary Fig. 5 for four select domains for visualization purposes; the full results are in Supplementary Table 8 (ref. ^17^)). Overall, we observed a high degree of variation unexplained by age or sex for a large fraction of proteins associated with CKMD in children and adults (example trajectories for key metabolic proteins LEP, IGFBP2, ACY1 and CES1 in Supplementary Fig. 5). Age or sex explained a relatively low proportion of variability for our traits with the notable exception of BMI-Z, which reflects growth and development. Of note, many proteins that showed a substantial variability not explained by age or sex changed with GLP1-RA therapy in adults (for example, IGFBP1, IL1RN, TNFSF14). This substantial, potentially modifiable residual variability in the CKMD-relevant proteome suggests potential targets for intervention to improve CKMD outcomes.

## Discussion

Climbing rates of CKMD-related morbidity over the past two decades have necessitated increasingly early screening and preventive measures to address population burden. Over the past four decades, evidence from early coronary artery pathology reports^1^, studies in Bogalusa^56^ and recent data in ≈36,000 children from the i3C Consortium^5^ have implicated obesity and related CKMD phenotypes (hypertension, pro-atherogenic dyslipidaemia, insulin resistance) as drivers of premature disease. In response, pharmacologic approaches^57^ and lifestyle interventions (for example, nutrition, physical activity) are increasingly emphasized in childhood to mitigate early CKMD risk. CKMD screening in children is triggered by the presence of obesity, which often misses at-risk children^58^. Furthermore, clinical intervention thresholds for blood pressure and lipids in children are less stringent than those used in adult care^3,5^, and standard screening tests (for example, fasting glucose) often miss a substantial amount of disease-defining risk in children^9,10^. Given that at-risk children generally do not suffer from clinical cardiovascular events, defining precise biomarkers in childhood that reflect relevant early CKMD traits and have prognostic and genetic links to CKMD outcomes in adults is critical to deploying surveillance and treatment strategies to prevent CKMD at its origin^57^.

Here, using complementary analyses across the paediatric CKMD phenome, circulating proteome and genome, we derived proteomic signatures of CKMD-related traits in children that showed notable concordance with an adult CKMD proteome. Composite, multi-protein signatures of CKMD risk in children were strongly associated with cross-sectional and incident CKMD clinical outcomes over a decade in ≈28,000 adults. The childhood CKMD proteome implicated both known and potentially novel biological mechanisms in CKMD. Proteins linked to CKMD traits in children showed (1) altered abundance following GLP-1RA therapy and (2) a potentially causal effect on adult CKMD outcomes (spanning cardiovascular, renal and metabolic diseases). Finally, proteins linked to CKMD in adults and children generally showed substantial variation not explained by age or sex in childhood through early adulthood, highlighting potentially modifiable components of early metabolic risk. These results are consistent with the presence of proteomic signals linked to adult CKMD beginning early in life, highlighting opportunities for use of the childhood proteome in enhanced precision risk assessment starting earlier in life.

Although overt clinical disease in childhood remains rare, classical risk factors for CKMD in adults also appear to promote early CKMD phenotypes in children: for example, reduced physical activity and low physical fitness levels promote insulin resistance^59^ and subclinical atherosclerosis in children^60^. Paediatric CKMD is also related to early myocardial tissue changes that presage heart failure^61^ as well as pro-atherogenic dyslipidaemia and inflammatory biochemical states ultimately linked to adverse outcomes in adulthood^5^. Given the limitations of standard clinical tests to capture this metabolic risk early (for example, fasting labs), there is growing recognition that molecular biomarkers—‘omics’—can potentially detect risk decades before disease onset and often independent of established clinical risk factors^14,62–66^. Our study contributes to emerging, considerable interest in addressing paediatric CKMD with findings that are broadly consistent with multiple published studies in CKMD (Extended Data Table 2). For example, in one of the largest studies to date in paediatric CKMD, 149 proteins (Olink) were quantified in 4,024 children in a cross-sectional analysis and 200 children during a 1-year obesity intervention^67^ (HOLBAEK study). In addition to identifying similar proteins in cross-sectional association with obesity status as in our study, proteins related to CKMD traits in our study showed changes in over 1 year that were associated with changes in CKMD traits in the HOLBAEK study, suggesting important phenome–proteome dynamicity of our findings. Furthermore, seminal work from the same group linking mass spectrometry proteomics in children to human genetics^68^ showed that circulating protein quantitative trait loci in children were linked to adult CKMD (analogous to our approach using adult proteogenomic models for proteins identified in paediatric CKMD).

Our results fundamentally extend previous findings via several key innovations: (1) we surveyed a broader proteomic space than previously quantified (Extended Data Table 2); (2) we focused on a high-risk paediatric population, with a high prevalence of obesity; (3) we included direct, imaging-based measures of MASLD, an early mediator of CKMD risk; and (4) we benchmarked paediatric proteomic signatures against adult outcomes and phenotypes, including adults from the same community as well as from a geographically and ethnically distinct population. Of note, the multisystem CKMD outcomes were most strongly associated with proteomic signatures of hepatic disease, a condition that affects nearly 10% of children^69^ and may serve as a harbinger of adult CKMD^70^. Moreover, we observed strong concordance in phenome–proteome relationships between youth and adults. Several proteins linked to paediatric CKMD mapped to known adult CKMD-relevant pathways across age groups, including beta-cell function and inflammation, as well as new molecular mechanisms of early CKMD biology. Finally, changes in protein abundance under GLP-1RA therapy, genetic associations with CKMD outcomes across the life-course and proteomic variation at the adolescent–adulthood transition not explained by age and sex provide strong support from external sources that these proteins mark phenotypic states in children that are biologically and therapeutically relevant.

The progression of cardiometabolic risk from childhood to adulthood has important clinical implications. With increasing pharmacological and surgical efforts to combat paediatric obesity (nearly 600% increase in paediatric GLP-1RA prescriptions between 2020 and 2023)^71,72^, a precision-based framework to prioritize youth at substantial CKMD risk to receive therapies will be critical to balance benefits with therapeutic and financial burden and clinical risk. Our identification of proteins in childhood CKMD that are predictive of adult CKMD and are associated with observed improvement via the GLP-1RA therapy in adults strongly supports the potential for precision proteomic risk assessments in paediatric CKMD. Nevertheless, our study has several limitations. The primary studied population was derived from a single population in South Texas. While consistent findings in the UK Biobank offer external validity, additional research in populations of diverse ancestries and socio-economic and lifestyle exposures is needed to extend generalizability and identify novel exposures and confounders relevant to proteomic prediction. In addition, our paediatric sample did not have serial sampling with power to detect dynamicity in the paediatric proteome over time, although we plan to continue to follow our cohort to accomplish this goal. Our results should also be viewed in the context of differences in proteomic platforms across data sources that may introduce variability, issues around detection specificity and subsequent bias. Nevertheless, our genetic findings (suggesting life-course implications), dynamicity with GLP-1RA in adults and, most importantly, concordance with large, very recent longitudinal studies in children, are strongly suggestive and support future longitudinal work. Dedicated serial studies (including under pharmacologic therapies, such as GLP-1) are needed to reaffirm the use of proteomic-based biomarkers for childhood metabolic health. Importantly, our proteomic approach identified biologically relevant effects with a smaller sample size than traditionally required for genetic analysis (thousands to over 1 million participants), probaby owing to greater biologic proximity of the proteome to measured phenotypes and outcomes. However, despite our ability to achieve clinically relevant effect estimates, certain potential confounders were not measured in sufficient numbers of participants and could not be adjusted for in our models, such as pubertal staging and genetic principal components in our paediatric cohort. Puberty is known to impact protein abundance; while we do have self-reported pubertal staging from the puberty development scale, most of our paediatric participants (72.5%) fall into the ‘pubertal’ stage, limiting our ability to explore effects in earlier stages of puberty. Finally, while we had insufficient data to adjust for paediatric genetic principal components in our LASSO models, our Cox models were robust to genetic principal component adjustment in the UK Biobank.

In summary, we characterized the proteomic architecture of CKMD in children living at the US–Mexico border across a range of cardiometabolic phenotypes, demonstrating similarities between the CKMD phenome–proteome relation across age, its biological plausibility, relevance to long-term clinical CKMD risk in adults through large cohort observational studies and human genetics, and mutability with GLP-1RA therapy. Our results support the hypothesis that the molecular profile of CKMD appears early in the life-course, with proteomic signals relevant to long-term clinical outcomes appearing even in childhood. In total, these observations underscore the potential for precision proteomic risk assessment in children to identify at-risk groups who may benefit from earlier preventive strategies aimed at improving CKMD-free longevity. Further work in large paediatric populations to understand modifiability is critical to clinical deployment of precision strategies to detect and forestall cardiometabolic complications that originate in childhood.

## Methods

### Study sample

Paediatric and parental or guardian assent and consent were obtained according to the approved institutional review board protocol (HSC-SPH-14-0141, UT Health Houston School of Public Health, Brownsville), including the use of optional bilingual instruments. Written informed consent was obtained from all participants under the approved institutional review board protocol.

This study used data from the Cameron County Hispanic Cohort, a substudy within the BHRC. This substudy is a longitudinal, population-based cohort study of individuals from randomly ascertained households from impoverished neighbourhoods in Cameron County, Texas, located on the US–Mexico border. Paediatric participants aged 8–17 were enrolled from participating BHRC households. Their study exams were included in the analysis if they were under 18 years at exam 1. Paediatric participants who reach age 18 are subsequently enrolled in the adult cohort study; for this analysis, when available, follow-up exams up to age 20 were included. Of the 607 BHRC youth exams potentially eligible for this analysis, 397 included serum proteomic profiling. Of these, 3 were excluded owing to poor sample quality, leaving a final analytic sample of 394 (273 individuals at exam 1 and 121 individuals at exam 2). For participants with 2 visits, the median time between paediatric exam 1 and paediatric exam 2 was 2.48 years (interquartile range = (2.22, 3.60)). Analyses for individual phenotypes include fewer exams for some traits based on data completeness (see Table 1 for total *N* per trait). All work in the analyses described herein was executed unblinded to the participants’ data.

Adults from the same community aged ≥18 are enrolled and followed longitudinally every 5 years. Of the 4,956 adult BHRC participants, 887 exams from 686 individuals had proteomics available and were included in analyses validating associations in adults; 494 participants were included for testing the fit of paediatric models to adult phenotypes. No participants included in the paediatric analyses were included in the internal adult replication; however, there were 46 first-degree relatives (parents or siblings).

### Paediatric and adult CKMD phenotyping

Descriptive statistics for each trait and outcome are reported in Table 1, with additional details available in Supplementary Table 1. Participants were examined in the Clinical Research Unit in Brownsville, Texas. Children were accompanied by a parent or guardian. Venipuncture was performed for fasting glucose, fasting insulin, HbA1c, lipid profile (HDL-C, TC and TG), creatinine, and alanine and aspartate aminotransferase (ALT, AST). From these measures, we derived HOMA-IR, calculated LDL-C, TG to HDL ratio, eGFR^73^, APRI and fibrosis-4 (FIB-4) index. Resting SBP and DBP were measured three times, with the average of the last two measures used, from which mean arterial pressure (MAP) and pulse pressure (PP) were calculated. Non-invasive ultrasound imaging of the liver using FibroScan (Echosens) was used to measure liver stiffness (kPA) and controlled attenuation parameter (CAP). Participants underwent whole-body dual energy X-ray absorptiometry for body composition via standard clinical protocols (Hologic) to derive the total and percentage fat mass, total and percentage lean mass (excluding bone mass), trunk fat and visceral fat area (cm^2^). Weight and height were measured by study staff for BMI. Age- and sex-specific BMI-Zs for children and adolescents were calculated based on the Centers for Disease Control and Prevention growth charts. Trait-specific quality control strategies are described in Supplementary Table 1.

### Proteomics

We measured 5,420 circulating proteins (Olink Explore HT) in 394 frozen serum samples from children and adolescents; three samples were removed owing to poor proteomic sample quality. Normalized protein expression (NPX; protein abundance in log_2_ units) was calculated as indicated by the manufacturer (version 6.7.2 Olink proprietary software). Methods for Olink-based proteome quantification have previously been described^74^. There were no missing NPX data; although some values did fall below the limit of detection, they were included in analyses to optimize discovery potential. In adults, we used the same method and panel for 509 exams from 495 participants (‘batch 2’) and an earlier panel (Olink Explore 3072 with 2,940 proteins) for 378 exams in 191 participants (‘batch 1’). Overlapping proteins were mapped across the two batches for *N*-weighted meta-analysis. The subset of 494 adult participants in batch 2 was used for comparisons of effect size.

### Association of the circulating proteome with CKMD phenotypes in children and adults

We used linear mixed-effects regression to estimate associations between proteins and CKMD phenotypes in children and adolescents. HOMA-IR and TG levels were log_10_- and log_2_-transformed, respectively, to ensure normality. All other variables were assumed to meet the assumptions of the models. We modelled each protein as a function of a single phenotype and included a participant-level random effect to account for repeated measurement of phenotypes over exams. In our primary analyses, we adjusted for age and self-reported sex. As a sensitivity analysis, we additionally adjusted each model for BMI-Z (or adult BMI), except for those with outcomes relating to body composition (for example, dual energy X-ray absorptiometry measures). Trait-specific modelling strategies are described in Supplementary Table 1. Type 1 error was controlled using the Benjamini–Yekutieli false discovery rate (FDR) across all trait–protein comparisons. In adults, we applied the same modelling strategy (and as genetic data were available for these participants, we additionally controlled for the first three genetic principal components) to measure the association between each phenotype and protein in adults (using standard deviation-scaled model coefficients). Effect sizes were compared with the paediatric results via Pearson correlation. Pathway analyses were performed using proteins associated with phenotypes (*q*-value < 0.05 in children), with all proteins used in the regression analysis as the universe background on Kyoto Encyclopedia of Genes and Genomes (KEGG) and WikiPathways (*q*-value < 0.05 for enrichment).

To assess curated evidence from published GWAS, we classified the phenotypes into six domains: dysglycaemia (fasting glucose, HbA1c, HOMA-IR), body composition (BMI-Z, total fat mass (TFM), total percentage fat (TPF), total lean mass excluding bone mass (TLM), total percentage lean mass excluding bone mass (TPM), trunk fat mass (TrF) and visceral fat area (VFA)), blood pressure (SBP, DBP, MAP, PP), dyslipidaemia (LDL-C, HDL-C, TC, TG, TG to HDL-C ratio), hepatic (ALT, AST, APRI, FIB-4 index, CAP, kPa) and renal (eGFR). We queried the top 30 proteins by effect size in the paediatric analyses associated with each of the six phenotype domains (*q*-value < 0.05; *n* = 245 unique proteins), along with proteins associated with at least one individual phenotype in children but not in adults (*n* = 109 proteins) in the Common Metabolic Diseases Knowledge Portal (CMDKP)^75^. From the CMDKP, we used the HuGE scores for all phenotypes to summarize extant evidence of the corresponding gene’s association with 22 CMDKP catalogued phenotype groups. Evidence was grouped into standard bins of compelling (HuGE score ≥ 350), extreme (≥ 100), very strong (≥ 30), strong (≥ 10), moderate (≥ 3), anecdotal (≥ 1) and no evidence (< 1) for visualization in heat maps^75^.

### Identifying a proteomic signature across CKMD phenotypes in childhood using machine learning approaches

We next investigated joint associations between the circulating proteome and 16 CKMD phenotypes across the 6 phenotype domains in 273 children with both proteomic and clinical data (Table 1). Our selected approach required a complete cross-sectional proteomic–phenotype dataset. Traits derived from linear combinations of other traits (for example, MAP and PP) were excluded from the PCA. Consequently, we excluded phenotypes analysed in the single-trait analyses that had over 50% missingness across the union of all participants in those analyses, leaving us with only 11 individuals with any missing data. We imputed missing phenotype data for those 11 individuals via random forest imputation (missForest). Before imputation, TG and HOMA-IR were log-transformed, all hepatic traits were inverse rank normalized, and all proteins and phenotypes were mean-centred and standardized.

We performed PCA across the 16 phenotypes, selected 6 principal components by scree plot evaluation and performed varimax post-rotation to generate LFs. Using each of the six LFs as a dependent variable and our proteomic data as the independent variables, we used L1-regularized regression with five-time repeated fivefold cross-validation (LASSO regression) to construct six parsimonious multivariable proteomic signatures. Model fit (*R*^2^) was evaluated by performance in cross-validation folds in caret.

### Paediatric proteomic signatures of CKMD and long-term adult CKMD outcomes

We next measured the relationship between the LASSO-derived proteomic scores for children (corresponding to each of the six LFs) and adult cross-sectional and longitudinal phenotype outcomes. We estimated the proteomic scores for each multi-phenotype LF in adults from two samples: (1) BHRC adults (*n* = 494) and (2) adults from the UK Biobank (*n* = 28,256)^76^. Given the differences in coverage across Olink platforms (Explore 3072 versus HT) between paediatric and adult samples (BHRC and UK Biobank used HT and Explore 3072, respectively), we ‘recalibrated’ our LASSO paediatric score for application across datasets, following published methods^11^. In brief, we refit each of our six models for the subset of proteins available on the Explore 3072 platform using the paediatric proteomic scores for each phenotypic LF as the dependent variable and paediatric proteins common across samples as independent variables. We confirmed adequate performance of the recalibrated LASSO model within children (*R*^2^ across LFs ranging from 0.72 to 0.98). Coefficients from the recalibrated LASSO-based LF proteomic models were applied to mean-centred and standardized protein abundance in the adult sample to generate adult LF proteomic scores for association analyses. LF proteomic scores were defined as the linear combination of the paediatric LASSO coefficient and the standardized protein values across all LASSO-included proteins. Standardized scores were then used as an independent variable in Cox regression models for all-cause mortality and selected CKMD outcomes.

Our analytic sample in the UK Biobank included 28,256 unique participants that had complete proteomics data for at least 1 of the scores generated in BHRC paediatric participants. From this full sample, we defined 6 different subsets of participants (with participants allowed to be in >1 subset) based on having complete data for each set of proteins in each LF-based proteomic score of paediatric CKMD. Clinical outcomes in the UK Biobank were defined using the International Classification of Diseases (ICD) codes summarized by the PheWAS package^77^. We excluded participants from models if a confounding ICD code was present or if they reported a prevalent diagnosis or related condition (UK Biobank Data Fields 2443, 20002). Time-to-death was defined by registry data (UK Biobank Data Field 40000), and non-deaths were censored on 30 November 2022. Time-to-event data for non-death outcomes were defined as the time to first qualifying ICD code, and non-event participants were censored at the date of death (if applicable) or region-specific dates (UK Biobank Data Field 54): 31 October 2022 for England, 31 July 2021 for Scotland and 28 February 2018 for Wales. Cox regression models included adjustments to capture metabolic risk measures used in clinical practice and more general confounders for mortality: age, sex, race and ethnicity, BMI, HbA1c, TG, HDL-C, Townsend deprivation index, smoking and alcohol use. We included non-linear terms as appropriate, given the potential non-linear dependence of CKMD risk on age. Our methods were adapted with minimal modification from previous work in our group to ensure scientific reproducibility^11^ (with attribution by this statement).

### Paediatric proteomic signatures related to dynamic changes in the circulating proteome with GLP-1 receptor agonist therapy in adults

Proteins associated with paediatric CKMD that are dynamic with GLP-1RA therapy may identify actionable biomarkers of long-term risk and support utilization of GLP-1RA in at-risk children. We mapped 1,032 proteins related to any CKMD phenotype in our paediatric sample (in age- and sex-adjusted models; *q*-value < 0.05) to a recently reported study of circulating proteins in the STEP 1 randomized clinical trial in adults (semaglutide)^15^. We examined changes in the circulating proteome after 68 weeks of treatment with semaglutide in the STEP 1 cohort (SomaScan aptamer-based platform), matching proteins identified in our cohort by UniProt identifier and restricting to those proteins with significant on-therapy change in STEP 1 (*q*-value ≤ 0.05). For duplicate aptamers for a given protein, the minimal absolute value effect size was chosen. The proteomic data from STEP 1 can be found in the supplemental data from a previous study^15^ (sheet ‘S2_tx_STEP1’, columns ‘effect_size’ and ‘qvalue’).

### Genetically predicted PWAS

We next investigated whether proteins implicated by paediatric CKMD phenotypes have relevance to disease development in adults, using genetic models for expression of these proteins in adults. To investigate the impact of paediatric CKMD-associated proteins on adult disease outcomes, we leveraged genetic prediction models derived from 7,213 European-ancestry participants with circulating proteomic data in the Atherosclerosis Risk in Communities (Somalogic aptamer-based proteomics)^78^. We conducted PWAS^16^ to identify proteins associated with adult CKMD outcomes, prioritizing those previously linked to paediatric CKMD phenotypes and responsive to GLP-1RA treatment (FDR < 5%). We integrated the protein expression models into the Mendelian randomization–joint tissue imputation pipeline to support causal inference^79^. Mendelian randomization–joint-tissue imputation incorporates gene expression prediction models and applies Mendelian randomization to estimate the causal effect of gene expression on complex traits. By leveraging multiple genetic instruments and cross-tissue information, it improves power and helps distinguish true causal genes from associations driven by linkage disequilibrium or co-expression; in the case of a single tissue, the method is equivalent to conventional PrediXcan. While a lack of large aptamer-based proteogenomic studies in childhood guided us to use adult genetic models of the proteome in this analysis, adult genetic models have been used to support the importance of lifelong exposure to key molecules in cardiovascular disease (for example, LDL and TG^80^).

Of the 245 proteins that were significantly associated with paediatric CKMD phenotypes and showed evidence of change after GLP-1RA based on STEP 1 trial findings, 131 proteins were available among the 1,318 elastic net genetic models of the proteome. We used these genetic models in concert with large-scale GWAS to test whether genetically predicted protein levels of these 131 proteins would be associated with CKMD clinical outcomes in adults, prioritizing key CKMD outcomes: (1) heart failure^81^ (*n* = 1,946,349 individuals from 42 studies, 153,174 cases), (2) BMI (*n* = 419,163 in the UK Biobank^82^), (3) T2D^83^ (*n* = 2,535,601 individuals, 428,452 cases), (4) renal function^84^ (creatinine; *n* = 1,004,040), (5) myocardial infarction^85^ (3,961 African American or Afro-Caribbean cases, 51,544 African American or Afro-Caribbean controls, 2,384 Hispanic or Latin American cases, 26,930 Hispanic or Latin American controls, 225 East Asian ancestry cases, 3,322 East Asian ancestry controls, 39,300 European ancestry cases, 276,368 European ancestry controls) and (6) MASLD^86^ (imaging *n* = 66,814 and diagnostic code (3,584 cases versus 621,081 controls)). We estimated the effect (*z*-score) of the genetically predicted protein levels on each clinical outcome as follows^87,88^:

$${Z}_{g}\approx {\sum }_{{l\in \mathrm{Model}}_{g}}{\omega }_{{lg}}\frac{\widehat{{\sigma }_{l}}}{\widehat{{\sigma }_{g}}}\frac{\widehat{{\beta }_{l}}}{{\rm{s.}}{\rm{e.}}(\widehat{{\beta }_{l}})}.$$

Here ${w}_{\mathrm{lg}}$ is the weight assigned to the variant *l* by the trained protein model; $\widehat{{\beta }_{l}}$ and s.e.($\widehat{{\beta }_{l}}$) are the GWAS effect size and corresponding standard error summary statistics, respectively; and $\widehat{{\sigma }_{l}}$ and $\widehat{{\sigma }_{g}}$ are the estimated variance for the variant *l* and protein *g*, respectively.

### Sources of variability in early development

To investigate sources of variability in early development, we used recent published data from a population-based birth cohort from Sweden (BAMSE)^17^. We extracted reported variance explained by age or sex and subject-specific random effect from linear mixed models analysing Olink Explore-derived NPX protein levels in 50 males and 50 females measured longitudinally at ages 4, 8, 16 and 24 (ref. ^17^). We plotted variance explained across variance components for proteins associated with both paediatric and adult CKMD in our BHRC sample. We highlighted a selection of participant-level proteins across time in BAMSE from the Human Protein Atlas (images taken directly from https://www.proteinatlas.org). Images for each protein were taken directly from the Human Protein Atlas (with credit by this statement for images included in Supplementary Fig. 5). Figures taken from the Atlas can be found at https://www.proteinatlas.org. An example URL is https://v25.proteinatlas.org/ENSG00000054356-PTPRN/blood#bamse.

### Reporting summary

Further information on research design is available in the Nature Portfolio Reporting Summary linked to this article.

## Supplementary information

Supplementary Information Supplementary Figs. 1–6.

Reporting Summary

Supplementary Tables Supplementary Tables 1–11.

## Source data

Source Data Fig. 2 Statistical source data.

Source Data Fig. 3 Statistical source data.

Source Data Fig. 4 Statistical source data.

Source Data Fig. 5 Statistical source data.

Source Data Fig. 6 Statistical source data.

Source Data Extended Data Fig. 1 Statistical source data.

Source Data Extended Data Fig. 2 Statistical source data.

Source Data Extended Data Fig. 3 Statistical source data.

Source Data Extended Data Fig. 4 Statistical source data.

## Data Availability

UK Biobank data used for this analysis is publicly available (https://www.ukbiobank.ac.uk) and was accessed using approved proposal 57492. Individual-level cohort data from the BHRC cannot be made publicly available owing to ethical constraints and restrictions imposed by the terms of participant informed consent. Raw proteomics data may be shared with qualified investigators for research purposes following review by the BHRC Project Review Committee (comprising internal and external advisors) and execution of a formal Data Use Agreement (DUA) with UTHealth Houston; these data are not available to the general public. Applications for data access can be initiated by contacting BHRCResearch@uth.tmc.edu. Source data are provided with this paper.

## Extended data

**Extended Data Table 1:** Demographic summary for adult participants analyzed from the UK Biobank

Characteristic	N	Value
Age (years)	28,256	58 (50, 64)
Female	28,256	15,273 (54%)
Ethnic Background	28,256	
Asian or Asian British		596 (2.1%)
Black or Black British		690 (2.4%)
Mixed		189 (0.7%)
Unknown | Other		495 (1.8%)
White		26,286 (93%)
Body mass index (kg/m^2^)	28,108	26.7 (24.1, 29.9)
Hemoglobin A1c (mmol/mol)	26,769	35.3 (32.9, 38.1)
Triglycerides (mmol/L)	26,839	1.47 (1.04, 2.14)
High-density lipoprotein (mmol/L)	24,599	1.40 (1.16, 1.68)
Smoking status	28,223	
Current		3,067 (11%)
Never | No answer		15,388 (55%)
Previous		9,768 (35%)
Alcohol use	28,223	
Current		25,753 (91%)
Never | No answer		1,371 (4.9%)
Previous		1,099 (3.9%)
Townsend Deprivation Index	28,218	-2.0 (-3.6, 0.8)
Whole body fat mass (kg)	2,425	3.0 (2.1, 5.2)
Whole body fat free mass (kg)	27,601	23 (18, 30)

Models for each protein score included a subset of participants with complete data required to generate the respective protein score. The number of participants for each model is as follows: LF1 = 22,389; LF2 = 22,607; LF3 = 24,504; LF4 = 25,018; LF5 = 22,814; LF6 = 21,955. Fat mass and fat-free mass were measured by bioimpedance. Continuous variables are reported as mean (25^th^ percentile, 75^th^ percentile), and categorical variables are reported as N (%).

**Extended Data Table 2:** Contextualization of findings to several recent studies in pediatric CKMD proteomics

Study	Design & proteomics platform	Contextualization to current findings
Goulding et al. (ALSPAC cohort)^99^	3005 children (age ≈9 years), Olink (96 proteins, inflammation)	• Analogous findings with inflammation (IL-6) to BMI and CKMD traits in children
Stinson et al. (HOLBAEK study)^67^	Cross-sectional: 4024 children (2377 with obesity; age 4-20)	• BMI-related proteins in HOLBAEK were similar to those found in association with CKMD traits in children (for example, LEP, CDCP1, HGF)
	Longitudinal: 200 children (age 7-17) with 1-yr obesity therapy	• Protein-BMI association in pediatric population was correlated with association in adults (UK Biobank), similar to findings in BHRC adults/children
	Olink (149 proteins)	• Changes in proteins related to CKMD (from our BHRC study) were associated with changes in cardiometabolic traits with weight loss in the Stinson et al. study (for example, AXIN1, CD40, CDCP1, CX3CL1, FGF21, HGF, IL10RB, IL18R1, IL7, LIFR, TNFSF14)
Krakowczyk et al. (meta-analysis)^100^	Meta-analyses of 20 studies in children for association with obesity (age 0-18; multiple assays used)	• Proteins implicated by CKMD in BHRC children have been shown in other cohorts in meta-analyses (for example, APOA1, ALDH members, IGFBP1, PTX3, UMOD, GSTA1, RBP4)
Stratakis et al. (HELIX project)^101^	863 children (mean age ≈7–8 years); multi-omics performed (Luminex for proteins)	• “High-risk” cluster for metabolic disease included proteins associated with CKMD in BHRC (for example, HGF, IL6)
Zhao et al.^102^	169 children (with and without obesity; mean age 11.3 years; mass spectrometry proteomics)	• Key proteins related to obesity also found associated with CKMD traits in BHRC (for example, SHBG, LEP, ADH1B, ADH4, IGFBP2, CHGA)
Paine-Cabrera et al.^103^	128 children (with and without MASLD; mean age ≈12–13 years; mass spectrometry proteomics)	• Key proteins related to MASLD also found associated with CKMD traits in BHRC (for example, SHBG, ADH4)
