# Supplementary material for: Paediatric proteomic signatures of cardiometabolic disease-associated traits predict adult disease outcomes
Source: Nat Metab. 2026 Sep 11;8(9):2007–22. doi: 10.1038/s42255-026-01589-7 (PMC13597307; doi:10.1038/s42255-026-01589-7)

nature metabolism

Supplementary information

https://doi.org/10.1038/s42255-026-01589-7

Paediatric proteomic signatures of cardiometabolic disease-associated traits predict adult disease outcomes

In the format provided by the authors and unedited

SUPPLEMENTARY FIGURES

Supplementary Figure 1. Protein correlation heatmap. Pairwise protein spearman correlations from the 245 unique proteins included in the circulating CKMD proteome (clustered).

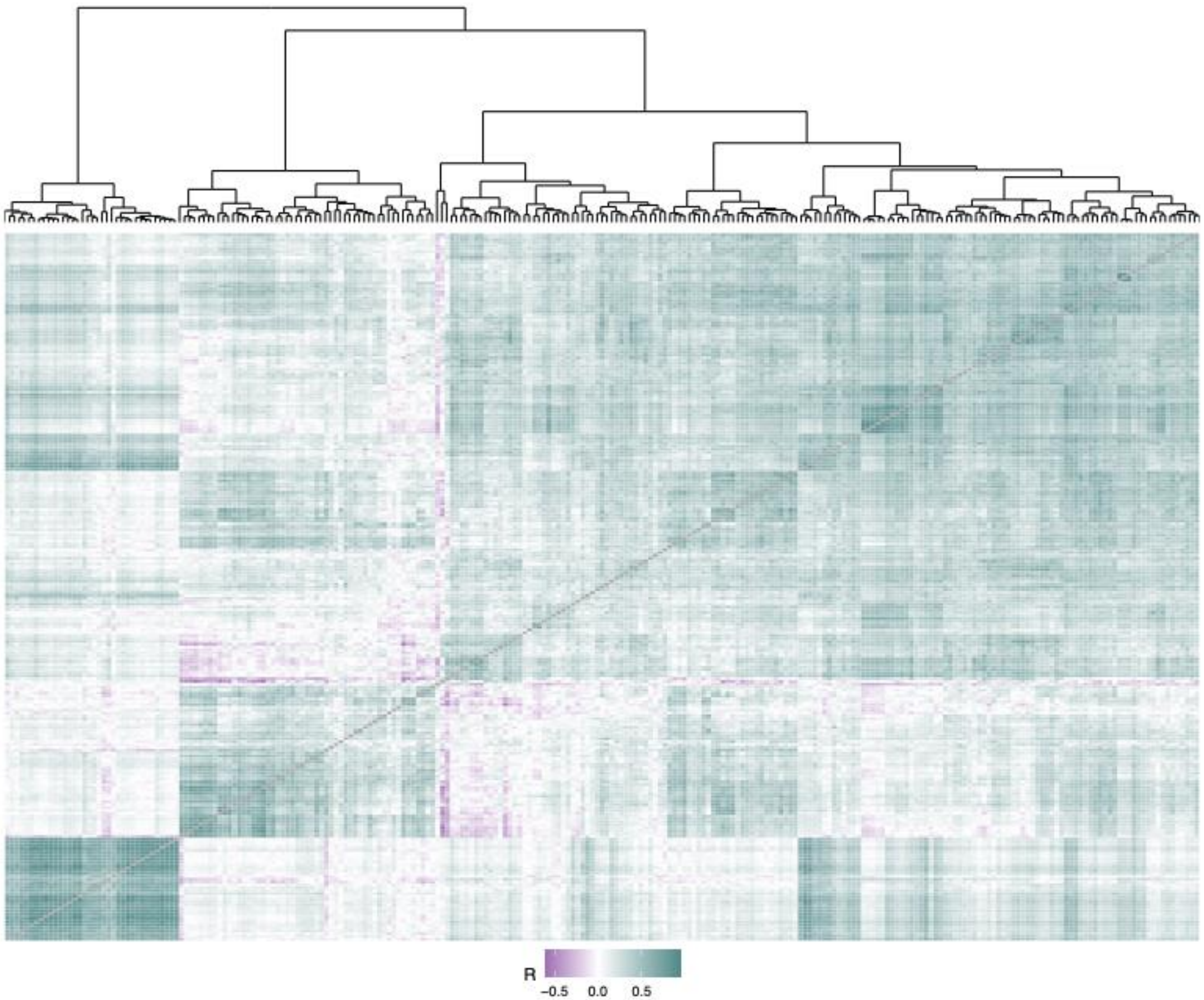

Supplementary Figure 2. Principal component scree plot. This scree plot indicates the eigenvalue of each component of the unrotated principal component analysis of the pediatric phenotype data. All components with eigenvalue > 1 (above the horizontal black line) were retained. Exact eigenvalues are available in Supplementary Table 4.

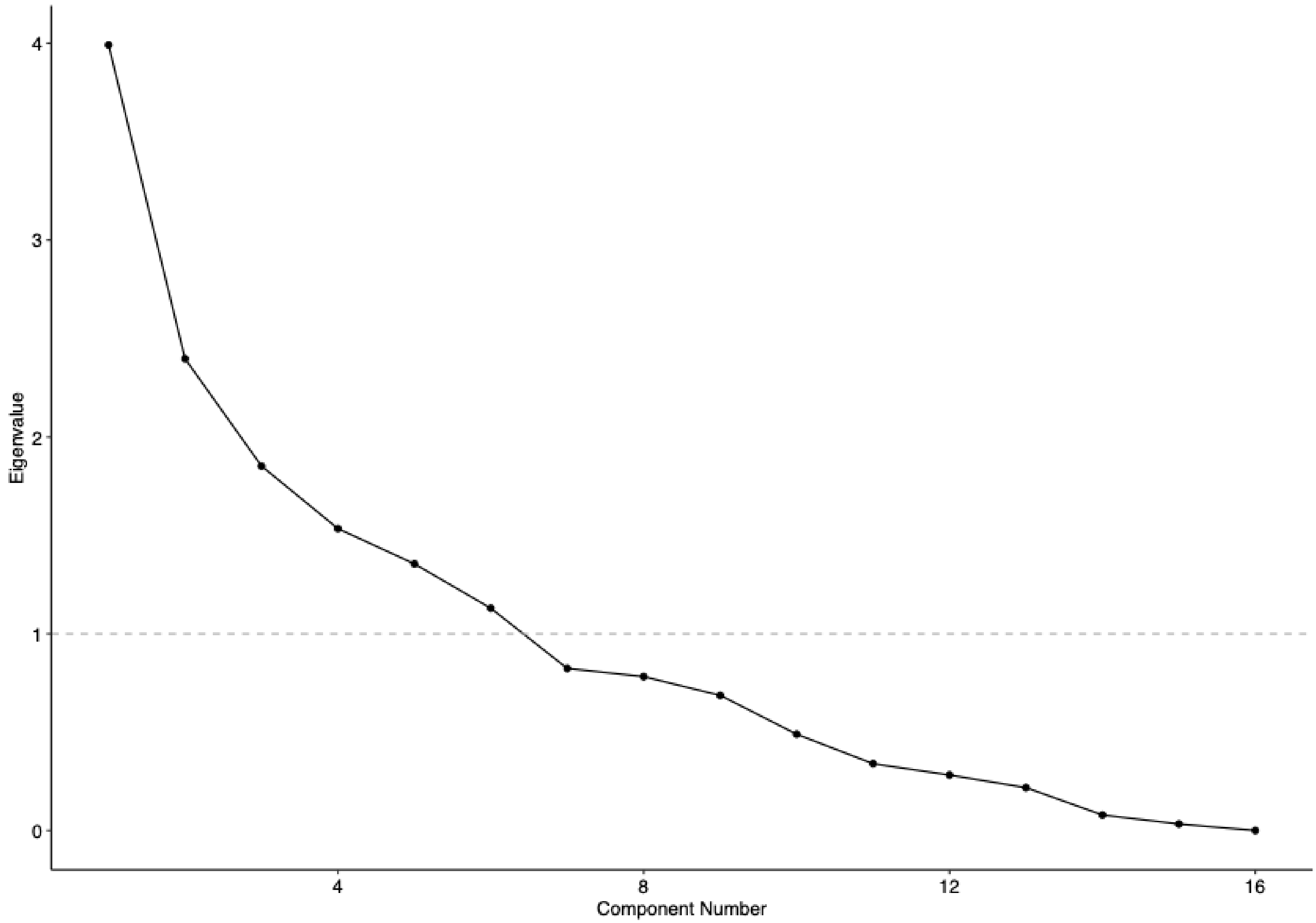

Supplementary Figure 3. LASSO feature importance. Top 50 coefficients by latent factor-based LASSO model sorted by feature importance (scaled absolute value of model coefficient). Full data are available in Supplementary Table 5.

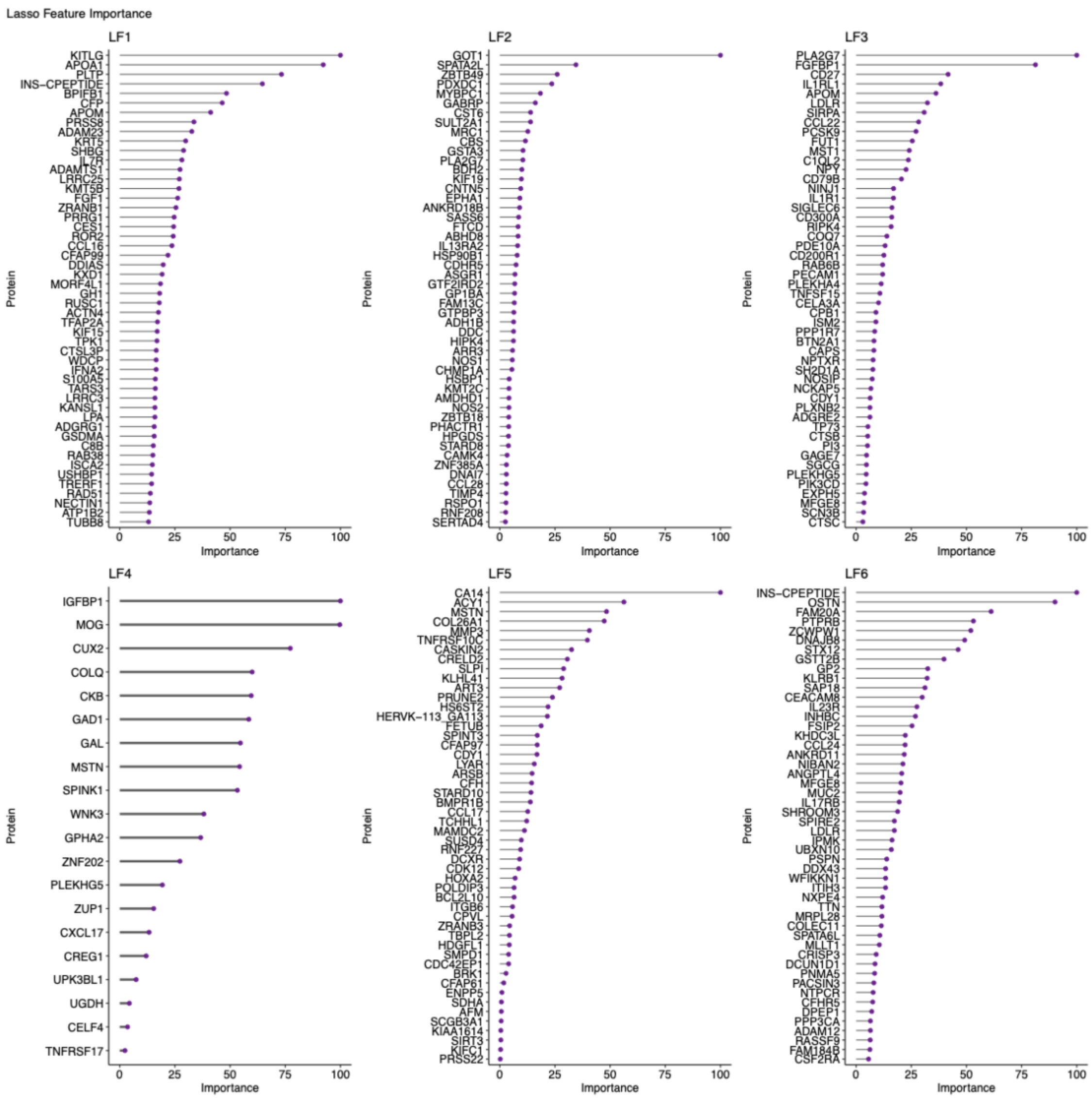

Supplementary Figure 4. Tissue and cellular localization of the pediatric CKMD proteome. Here, the "pediatric CKMD proteome" refers to proteins related to CKMD phenotypes in children that are also show changes in abundance with GLP-1RA therapy (both at 5% FDR, see **Methods**). Panel (A) displays tissue RNA-seq, demonstrating representation across liver, heart, adipose, kidney, and brain. Panel (B) displays a UMAP representation of *Tabula sapiens*, demonstrating transcriptional activity across different cell/tissue types. Panel (C) demonstrates tissue- and cell-specific transcriptional activity, highlighting similar tissues as (A) and specifically hepatocytes, pancreatic and renal cell types.

A

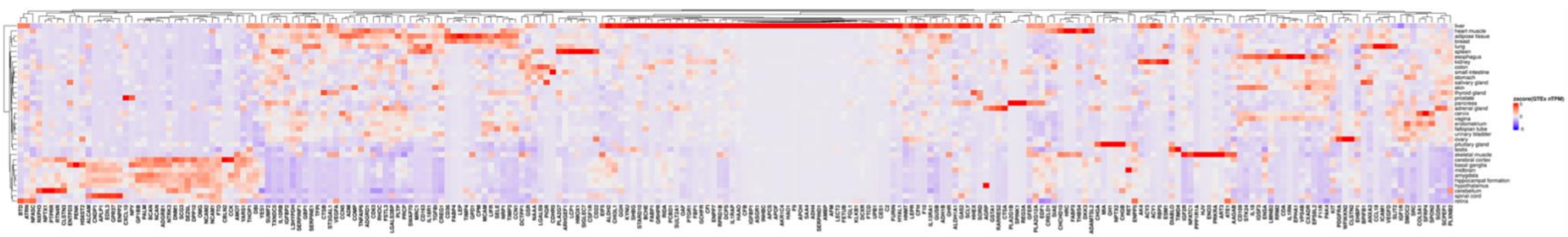

B

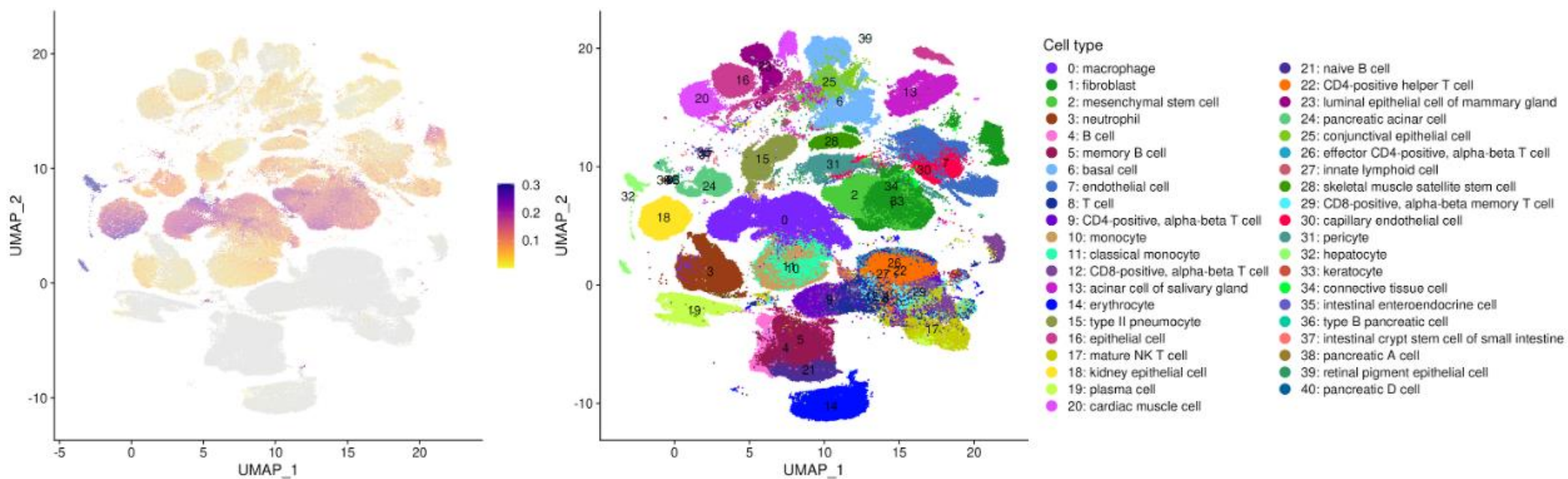

C

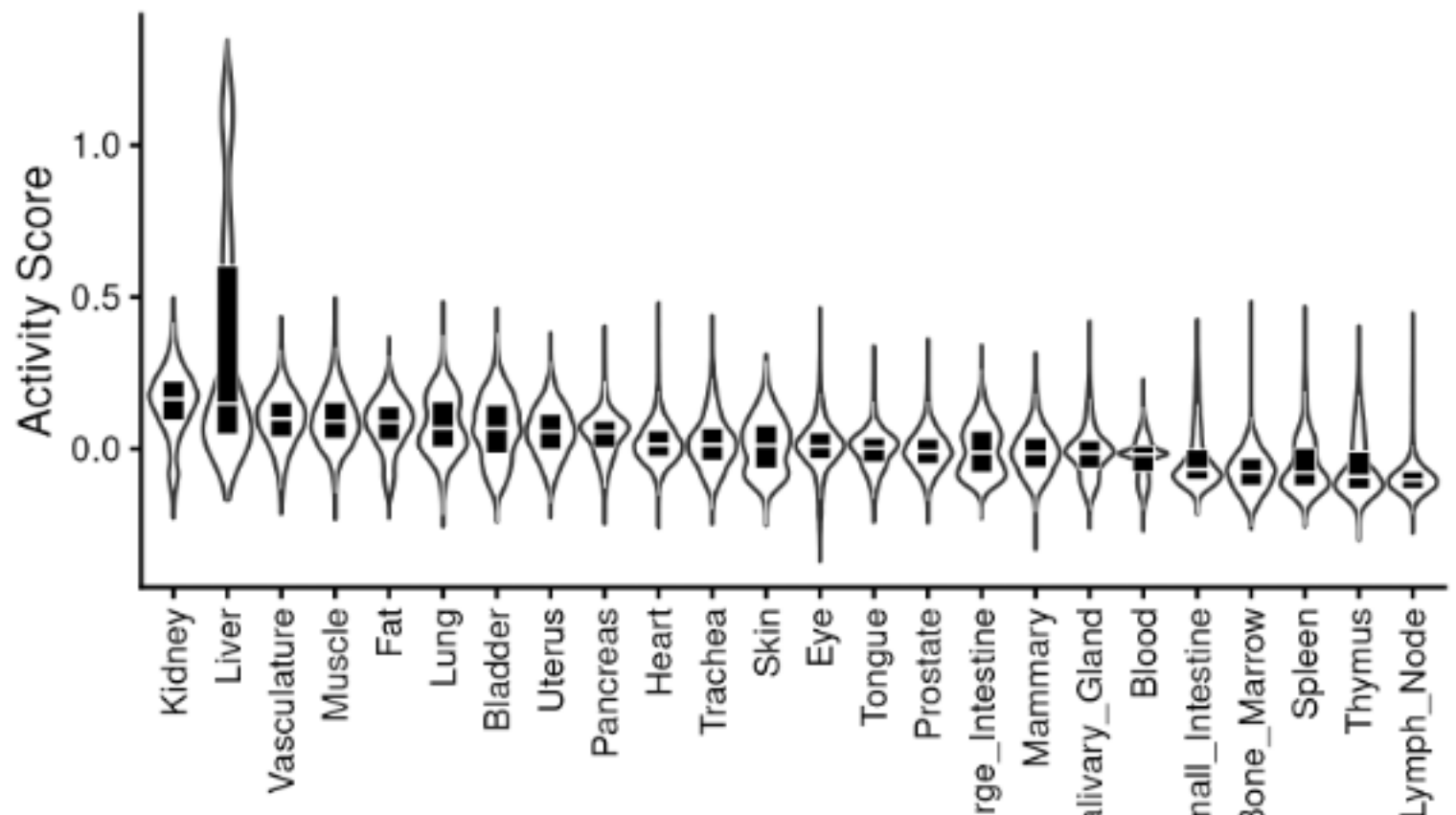
Activity Score
1.0
0.5
0.0
Kidney
Liver
Vasculature
Muscle
Fat
Lung
Bladder
Uterus
Pancreas
Heart
Trachea
Skin
Eye
Tongue
Prostate
Large_Intestine
Mammary
Salivary_Gland
Blood
Small_Intestine
Bone_Marrow
Spleen
Thymus
Lymph_Node

D

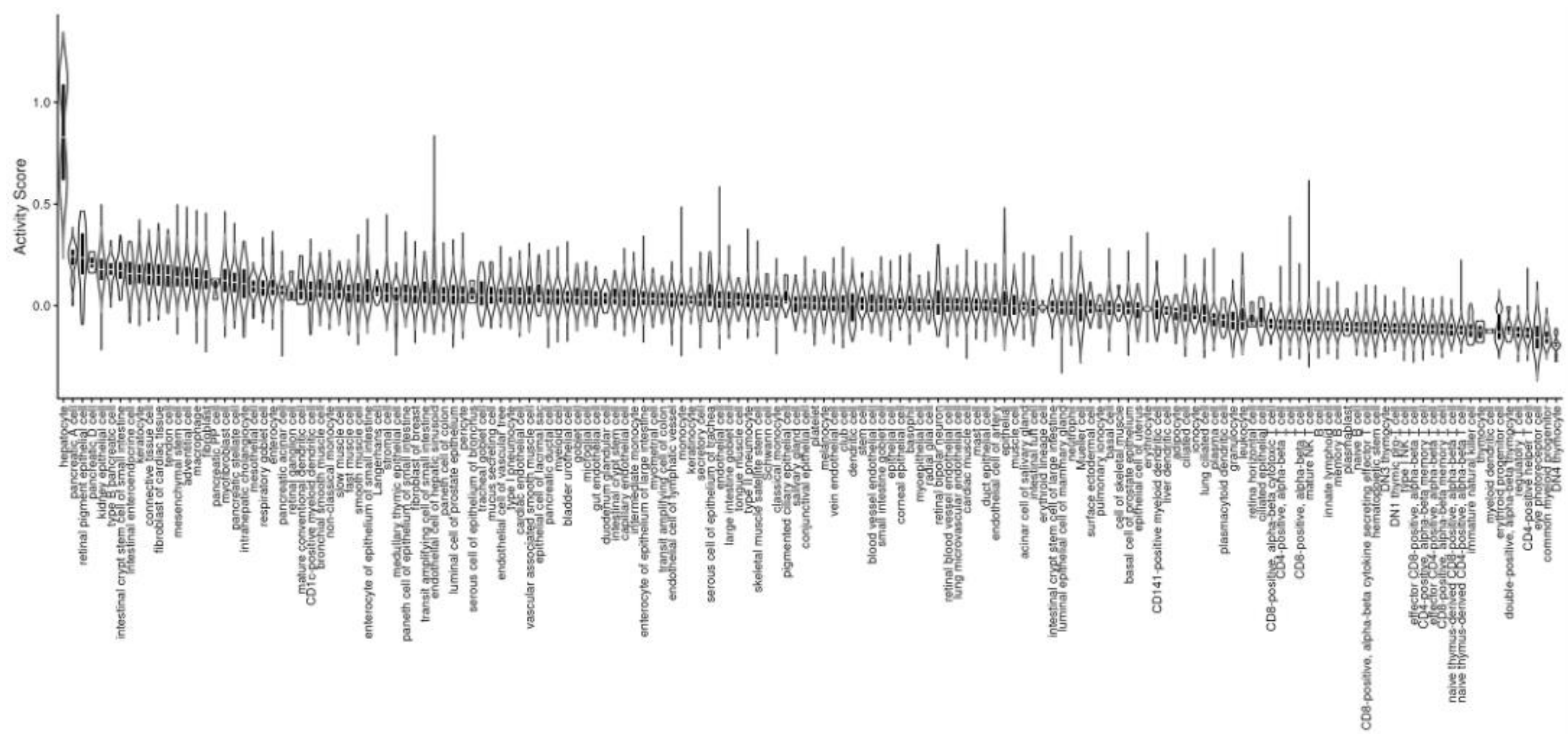
Activity Score
1.0
0.5
0.0

Supplementary Figure 5: Variation of the CKMD proteome during early development. Top panel visualizes variance components in 100 children from the Swedish BAMSE cohort as reported recently in Alvez et al.[17] for 6 phenotypes (BMI Z score, liver CAP, eGFR, HOMA-IR, triglyceride, and HDL). The outer band defines the phenotype. The middle band is a bar plot that shows the variance components (age/sex, per subject random effect, and residual) for each protein. The inner band shows the association effect size (beta coefficient) in BHRC children and adults. Red gene names indicate genes that were significant across multiple phenotypes. Regression coefficients for BHRC children and adults are scaled. The protein plots under the circus visualization depict protein levels (NPX) across early development to young adulthood from the Human Protein Atlas (see Methods). The top two proteins (PTPRN and IGFBP6) are included as examples of high variation explained by age and sex. Remaining proteins are examples across the spectrum of variance explained.

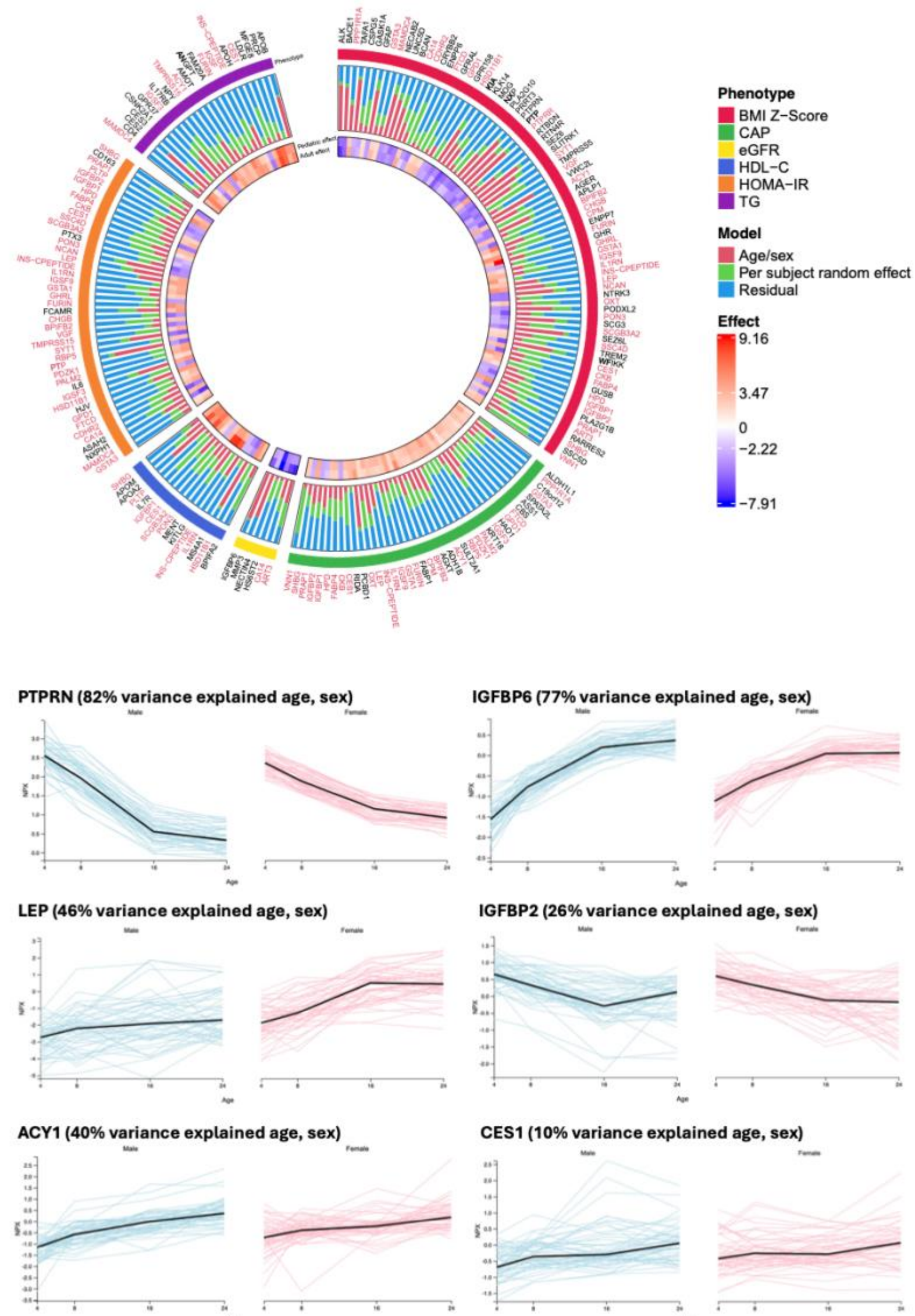

Supplement: Supplementary file 1 [file 42255_2026_1589_MOESM1_ESM.pdf]
