# Supplementary material for: Paediatric proteomic signatures of cardiometabolic disease-associated traits predict adult disease outcomes
Source: Nat Metab. 2026 Sep 11;8(9):2007–22. doi: 10.1038/s42255-026-01589-7 (PMC13597307; doi:10.1038/s42255-026-01589-7)
Supplement: Supplementary file 2 [file 42255_2026_1589_MOESM2_ESM.pdf]

nature portfolio

Corresponding author(s): Ravi Shah, Kari North

Last updated by author(s): Jul 6, 2026

Reporting Summary

Nature Portfolio wishes to improve the reproducibility of the work that we publish. This form provides structure for consistency and transparency in reporting. For further information on Nature Portfolio policies, see our Editorial Policies and the Editorial Policy Checklist.

Statistics

For all statistical analyses, confirm that the following items are present in the figure legend, table legend, main text, or Methods section.

n/a	Confirmed	
☐	☒	The exact sample size (*n*) for each experimental group/condition, given as a discrete number and unit of measurement
☐	☒	A statement on whether measurements were taken from distinct samples or whether the same sample was measured repeatedly
☐	☒	The statistical test(s) used AND whether they are one- or two-sided<br>*Only common tests should be described solely by name; describe more complex techniques in the Methods section.*
☐	☒	A description of all covariates tested
☐	☒	A description of any assumptions or corrections, such as tests of normality and adjustment for multiple comparisons
☐	☒	A full description of the statistical parameters including central tendency (e.g. means) or other basic estimates (e.g. regression coefficient) AND variation (e.g. standard deviation) or associated estimates of uncertainty (e.g. confidence intervals)
☐	☒	For null hypothesis testing, the test statistic (e.g. *F*, *t*, *r*) with confidence intervals, effect sizes, degrees of freedom and *P* value noted<br>*Give P values as exact values whenever suitable.*
☒	☐	For Bayesian analysis, information on the choice of priors and Markov chain Monte Carlo settings
☐	☒	For hierarchical and complex designs, identification of the appropriate level for tests and full reporting of outcomes
☐	☒	Estimates of effect sizes (e.g. Cohen's *d*, Pearson's *r*), indicating how they were calculated

Our web collection on statistics for biologists contains articles on many of the points above.

Software and code

Policy information about availability of computer code

Data collection	Olink's Explore v6.7.2 calculation module for proteomics assays, and body composition was measured via dual-energy X-ray absorptiometry (DXA) using Hologic's Horizon W APEX.
Data analysis	Data analysis for this manuscript was performed using a combination of Python 3, R 4, and SAS 9.4.

For manuscripts utilizing custom algorithms or software that are central to the research but not yet described in published literature, software must be made available to editors and reviewers. We strongly encourage code deposition in a community repository (e.g. GitHub). See the Nature Portfolio guidelines for submitting code & software for further information.

Data

Policy information about availability of data

All manuscripts must include a data availability statement. This statement should provide the following information, where applicable:

- Accession codes, unique identifiers, or web links for publicly available datasets
- A description of any restrictions on data availability
- For clinical datasets or third party data, please ensure that the statement adheres to our policy

The primary data used for this study is available upon reasonable request of the BHRC leadership, subject to satisfying data protection requirements of the IRB and participant consents.

Research involving human participants, their data, or biological material

Policy information about studies with human participants or human data. See also policy information about sex, gender (identity/presentation), and sexual orientation and race, ethnicity and racism.

Reporting on sex and gender	All analyses in this manuscript, where applicable, controlled for participant sex, as gender was not recorded in the clinics where examinations were conducted.
Reporting on race, ethnicity, or other socially relevant groupings	All BHRC participants in this study self-identified, broadly, as Hispanic/Latino; we therefore did not further adjust for ethnicity in our analyses. We did not include race as a covariate in any of our BHRC-related analyses, but we did report ethnic background summary statistics for our UK Biobank cohort.
Population characteristics	Our study consisted mainly of Hispanic/Latino pediatric participants age 8-20. We report summary statistics for all measured lab values and clinical classifications in Table 1, and we indicate whether those classifications are used clinically for children or for adults. We also included data from an adult cohort of participants in this study, the statistics for which are also found in Table 1. Additionally, for our UK Biobank analyses, we report all summary statistics, including ethnic background, in Table 3.
Recruitment	BHRC participants for this study were recruited via recruitment within census tracts in Brownsville, TX, and households within each tract were randomly invited to participate in the study.
Ethics oversight	Pediatric assents/consents and parental/guardian consents were obtained according to the approved IRB protocol (HSC-SPH-14-0141, UT Health Houston School of Public Health-Brownsville), including the use of optional bilingual instruments. Written informed consent was obtained from all participants under the approved IRB protocol.

Note that full information on the approval of the study protocol must also be provided in the manuscript.

Field-specific reporting

Please select the one below that is the best fit for your research. If you are not sure, read the appropriate sections before making your selection.

☒ Life sciences ☐ Behavioural & social sciences ☐ Ecological, evolutionary & environmental sciences

For a reference copy of the document with all sections, see nature.com/documents/nr-reporting-summary-flat.pdf

Life sciences study design

All studies must disclose on these points even when the disclosure is negative.

Sample size	Sample size is detailed in depth in both Figure 1 and Table 1 for all BHRC analyses; for UK Biobank analyses, sample size is included in both Table 3 and the manuscript. Because we used the largest sample available to us subject to data availability, no calculations were performed to determine its size. For our pediatric cohort, we had 273 unique participants in the study; for our adult cohort, we had 494 unique adults. For the UK Biobank analyses, we had n = 28,256 participants.
Data exclusions	All exclusion criteria, where predetermined, are specified in Supplementary Table 1. Variables excluded due to significant missingness in our multi-trait analysis or poor sample quality are indicated in the Methods section of our manuscript.
Replication	There was no replication analysis conducted. Model validation was performed internally using our adult population and in UK Biobank on a distinct cohort of study participants.
Randomization	Study participants were randomly sampled, but there were no experimental groups necessary for our non-experimental study design.
Blinding	Because there were no experimental groups, blinding was not applicable to this study.

Reporting for specific materials, systems and methods

We require information from authors about some types of materials, experimental systems and methods used in many studies. Here, indicate whether each material, system or method listed is relevant to your study. If you are not sure if a list item applies to your research, read the appropriate section before selecting a response.

Materials & experimental systems

n/a	Involved in the study
☒	☐ Antibodies
☒	☐ Eukaryotic cell lines
☒	☐ Palaeontology and archaeology
☒	☐ Animals and other organisms
☒	☐ Clinical data
☒	☐ Dual use research of concern
☒	☐ Plants

Methods

n/a	Involved in the study
☒	☐ ChIP-seq
☒	☐ Flow cytometry
☒	☐ MRI-based neuroimaging

Plants

Seed stocks	*Report on the source of all seed stocks or other plant material used. If applicable, state the seed stock centre and catalogue number. If plant specimens were collected from the field, describe the collection location, date and sampling procedures.*
Novel plant genotypes	*Describe the methods by which all novel plant genotypes were produced. This includes those generated by transgenic approaches, gene editing, chemical/radiation-based mutagenesis and hybridization. For transgenic lines, describe the transformation method, the number of independent lines analyzed and the generation upon which experiments were performed. For gene-edited lines, describe the editor used, the endogenous sequence targeted for editing, the targeting guide RNA sequence (if applicable) and how the editor was applied.*
Authentication	*Describe any authentication procedures for each seed stock used or novel genotype generated. Describe any experiments used to assess the effect of a mutation and, where applicable, how potential secondary effects (e.g. second site T-DNA insertions, mosiacism, off-target gene editing) were examined.*
